# Base-Promoted Synthesis of β-Substituted-Tryptophans via a Simple and Convenient Three-Component Condensation of Nickel(II) Glycinate

**DOI:** 10.3390/molecules22050695

**Published:** 2017-04-27

**Authors:** Rui Zhou, Zhaoping Pan, Yuehua Zhang, Fengbo Wu, Qinglin Jiang, Li Guo

**Affiliations:** 1 West China School of Pharmacy, Sichuan University, Chengdu 610041, Sichuan, China; sklb_zhourui@126.com (R.Z.); phapan@163.com (Z.P.); 2 Department of Medicinal Chemistry, School of Pharmacy, Chengdu Medical College, Chengdu 610500, Sichuan, China; 3 Department of Pharmacy, West China Hospital, Sichuan University, Chengdu 610041, Sichuan, China; 2016224060001@stu.scu.edu.cn (Y.Z.); wufengbo_@163.com (F.W.)

**Keywords:** three-component reaction, nickel(II) glycinate, β-substituted-tryptophans, convenient

## Abstract

A three-component reaction of nickel(II) glycinate was conducted for the convenient synthesis of β-substituted-tryptophans. The reaction worked smoothly under mild conditions and the procedure was simple and easy to handle.

## 1. Introduction

Non-canonical amino acids, both natural and unnatural, have been applied in the development of chemical probes and asymmetric catalysts, as well as industrial products and small-molecule pharmaceuticals [1,2,3,4]. The replacement of natural amino acids by unnatural amino acids could be considered as a way of investigating peptide-receptor relationships [5,6,7,8]. Tryptophan is an essential amino acid for many organisms. Similar to tryptophan, β-substituted tryptophan analogs are important building blocks of many bioactive compounds and natural products, such as celogentin C [9,10,11,12,13], stephanotic acid [14,15,16], hemiasterlin [17,18], milnamide A [19,20,21], and other alkaloids.

**Figure 1:**
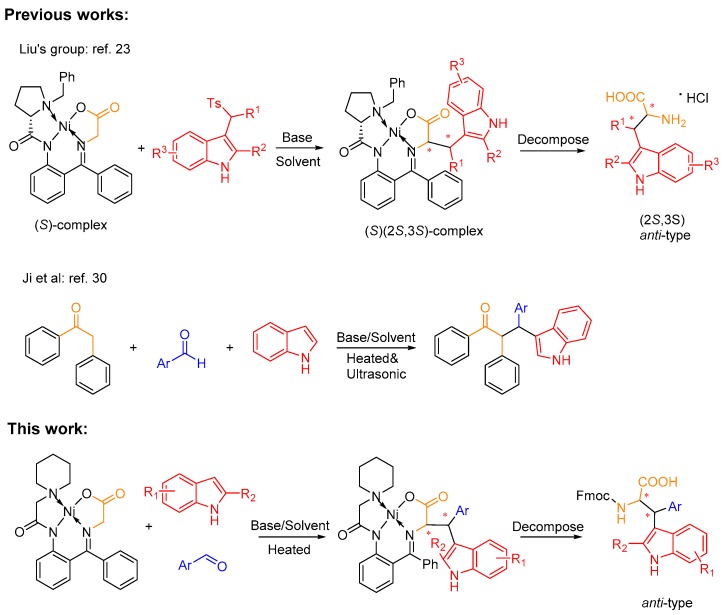
Three-component condensation based on previous works.

The synthesis of β-substituted-tryptophans has always been an interest to researchers. Previously, Hou et al. carried out the reaction of glycine derivatives with sulfonylindoles in the presence of catalytic AgCl and a chiral monodentate phosphoramidite to efficiently synthesize the *anti*-β-substituted-tryptophans [22]. Liu’s group used the chiral nickel(II) complex of glycine Schiff base to obtain the *anti*-β-substituted-tryptophans via an asymmetric Michael addition (Figure 1) [23] (the author claimed that they had obtained an *syn*-type product (*S*)(2*S*,3*R*). However, the crystal structure actually provided should be determined as (*S*)(2*S*,3*S*), indicating an *anti*-type product. The author seemed to have made a mistake about it). Arnold et al. used an engineered subunit of tryptophan synthase for the biosynthesis of β‑branched tryptophan analogues [24].

Three-component reactions of indoles, aldehydes, and an active methylene have been widely reported for their efficiency in construction of different indole-containing scaffolds [25,26,27,28,29]. These condensations were almost all conducted under protic or Lewis acid conditions. Ji et al. have performed pioneering work on base-promoted synthesis of β-indolylketones via a similar reaction (Figure 1) [30].

Reactions of nickel(II) complexes are notable for the use of readily-available and cost-effective procedures and many impressive works have been done using nickel(II) complexes, especially in V.A. Soloshonok’s and H. Liu’s groups [31,32,33,34,35,36,37,38].

**Scheme 1:**
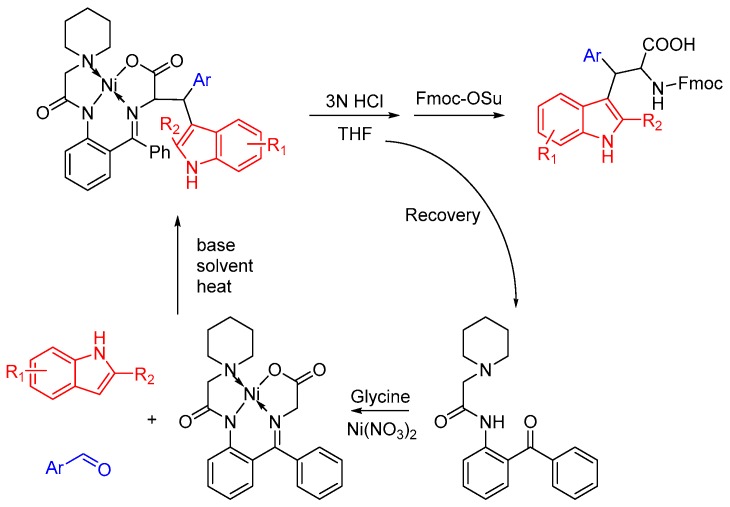
Base-promoted synthesis of β-substituted-tryptophans via a simple and convenient three-component condensation.

Considering the advantages of nickel(II) complexes in the synthesis of β-substituted amino acids, here we report a simple and convenient three-component reaction of nickel(II) glycinate with indoles and aldehydes under base-promoted conditions. A Nickel(II) complex is used in this type of three-component condensation for the first time, and this piece of work is highlighted for: (1) being a simple procedure, easy to handle for beginners; (2) mild conditions widely reachable for most labs; and (3) success in obtaining Fmoc-protected β-substituted tryptophan (Scheme 1).

## 2. Results and Discussion

**Figure 2:**
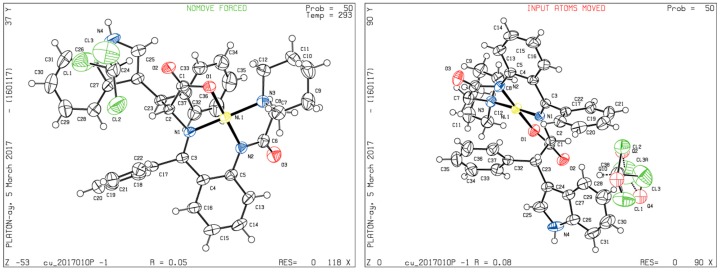
Relative configuration of **4a** from different angles.

As most of these kinds of reactions were conducted in acidic conditions, indole, benzaldehyde, and nickel(II) glycinate were first reacted together with the catalysis of acids. It turned out that this reaction could not progress under acidic conditions, but the target product was found when a strong base was used. Since commonly-used chiral nickel(II) glycinate generated very complicated products in this reaction under strong base conditions, the nickel(II) complex of glycine Schiff base with *N*-(2-benzyoly-phenyl)-2-piperidino-acetamide was used for the optimization of the reaction conditions. Protic solvents were preferred and glycerol seemed to be the best solvent (Table 1, Entry 3). Several bases were tested to see if there would be any improvement of this reaction, including LiOH, ethyldiisopropylamine (DIEA), 1,5-diazabicyclo[5.4.0]-5-undecene (DBU), and 1,1,3,3-tetramethylguayyniaine (TMG). Temperature also served as an important condition and the reaction could not progress while the temperature was below 50 °C (Table 1, Entry 9). The most noticeable drawback of this reaction appeared to be the moderate yield. In fact, besides the desired product obtained as a single diastereo-isomer, an orange solid consisting of 30–40% the total mass was also obtained. This solid was of bad solubility to many solvents and we failed to obtain a clear enough NMR spectrum for its identification. MS study of this solid gave no desired *m*/*z*+ peak, indicating that this solid might not be the other diastereo-isomer. Thus, we assumed that the reaction was of good diastereo-selectivity and the yield here was actually for a single diastereo-isomer. The relative configuration of **4a** was detected to be (*R*,*R*) or (*S*,*S*) by X-ray crystallography (CCDC 1530835; CCDC 1530835 contains the supplementary crystallographic data for this paper. These data can be obtained free of charge via http://www.ccdc.cam.ac.uk/conts/retrieving.html (or from the CCDC, 12 Union Road, Cambridge CB2 1EZ, UK; Fax: +44-1223-336033; E-mail: deposit@ccdc.cam.ac.uk)). It means that we got an *anti*-type product (Figure 2).

Various aromatic aldehydes and substituted indoles were used to investigate the substrate scope of this reaction under the optimized conditions (Table 2). We can see from Table 2 that substitution on either benzaldehyde or indole slightly affected the reaction. Most of the substrates could take part in the reaction to give the products in moderate yields. The nitro group at the *para*-position resulted in the lowest yield, while methyl on the *para*-position resulted in the highest. Introduction of 4-(dimethylamino)benzaldehyde and pyridine-4-aldehyde could not give the corresponding products and yet we do not know why. Notably, introduction of ferrocenecarboxaldehyde afforded the corresponding product smoothly. Ferrocene have been reported to have special bio-functions in drug delivery and antigen detection [39,40,41,42] and ferrocene-containing amino acids could be convenient building blocks. The structures proposed for all products were in agreement with their NMR spectra. The relative configuration of these structures should be the same as **4a**, for the δ values and *J* values of the protons on adjacent stereocenters were similar among the ^1^H-NMR spectra of compounds **4a**–**4n**. NOE spectra of **4a** and **4j**, which could be considered as assisting data, were given in the Supplementary Materials (Figure S2). The results above corresponded to the results of X-ray analysis.

**Figure 3:**
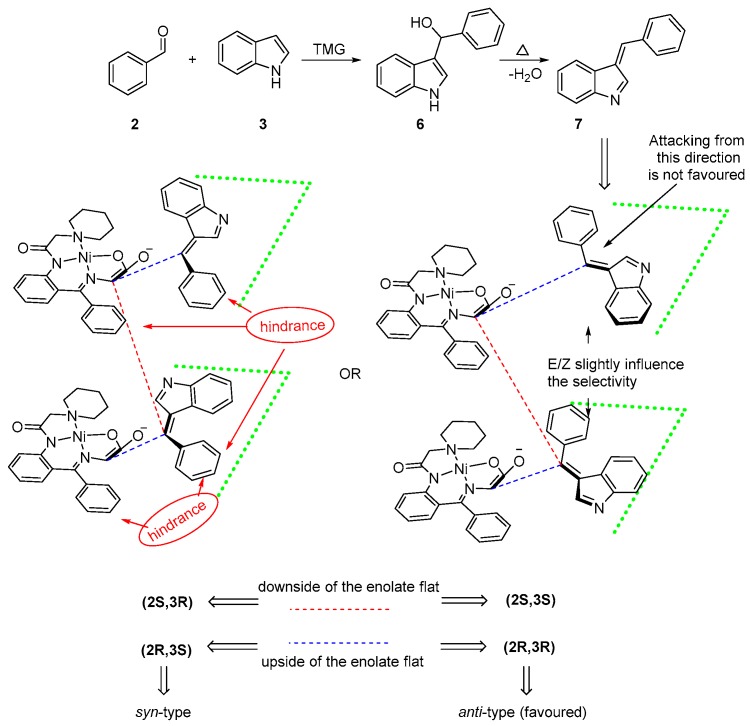
Plausible mechanism for the reaction.

**Scheme 2:**
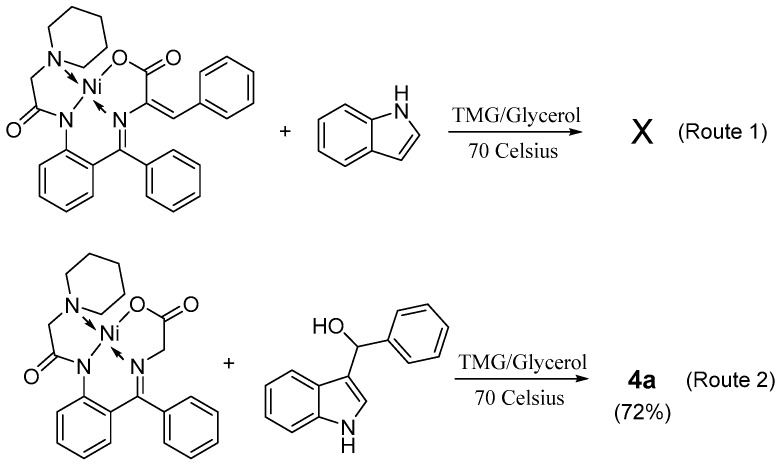
Reaction of Knoevenagel product and indole (Route 1) gave no desired product; reaction of complex and 3-indolyphenylmethanol (Route 2) gave the product **4a** smoothly.

A plausible mechanism for the reaction could be explained as the follows: reaction of aldehyde **2** and indole **3** occurred under the strong base condition to form 3-indolyl-arylmethanol **6**, which dehydrated while heated to form intermediate **7**. Sequential Michael addition of intermediate **7** and nickel(II) glycinate gave the desired product (Figure 3). To further confirm the reaction process, relevant reactions were conducted under the same conditions. The results were corresponding to previous study (Scheme 2) [30].

The diastereo-selectivity of this reaction should be explained as follows (Figure 3): intermediate **7** formed a flat structure and the double-bond of the enolate formed another flat structure, which was restricted by the complex. Conjugation of the two flat structures could happen from certain directions as shown in Figure 3, leading to different configurations. The relative configuration was altered when one of the two flats was flipped. The *syn*-type configurations were not favored mainly because of the hindrance between the phenyl groups. The E/Z of intermediate **7** might slightly influence the preference of configurations, but not definitively.

We compared our crystal structure with the one in previous work [23] learning that we had the same relative configuration. However, there was an obvious conformational difference, which might be related to the E/Z of intermediate **7**. The results indicated that the above explanation makes sense.

The decomposition of the complex **4** could result in the obtaining of the β-substituted-tryptophans, and the ligand could be recovered and reused via a simple procedure according to previously reported methods [23]. Herein, we provided an example, as in most previous works, in which Fmoc-β-substituted-tryptophan **5a** was obtained by in situ Fmoc-protection after the decomposition of the complex **4a**.

## 3. Materials and Methods

### 3.1. General

The reagents (chemicals) were purchased from commercial sources, and used without further purification. Analytical thin layer chromatography (TLC) was GF254 (0.15–0.2 mm thickness). The mass spectra and high resolution mass spectra were obtained using Waters TOF-MS instrument (Waters, Milford, MA, USA). The ^1^H- and ^13^C-NMR spectra have been respectively measured in CDCl_3_ or DMSO-*d_6_* at 400 and 100 MHz using a Bruker Avance III 400 MHz instrument (Bruker BioSpin GmbH, Rheinstetten, Germany) with TMS as an internal standard.

### 3.2. Typical Procedure for the Synthesis of Nickel(II) Complex (***1***)

This procedure is according to previous work [43]. A solution of potassium hydroxide (9 equiv.) in methanol was added to a suspension of *N*-(2-benzyoly-phenyl)-2-piperidino-acetamide (1 equiv.), glycine (5 equiv.) and nickel nitrate hexahydrate (2 equiv.) in methanol at 60–70 °C. Upon complete consumption of the *N*-(2-benzyoly-phenyl)-2-piperidino-acetamide, the reaction mixture was poured into icy 5% acetic acid solution. The precipitation was filtered and washed with water, then dried. The product obtained was in high chemical purity for further use.

### 3.3. General Procedure for the Synthesis of Nickel(II) Complex (***4a**–**n***)

The Nickel(II) complex of glycine **1** (0.2 mmol), aromatic aldehydes **2** (0.4 mmol), and indoles **3** (0.4 mmol) were suspended in glycerol (2 mL), then tetramethylguanidine (TMG, 0.6 mmol) was added. The reaction mixture was heated to 70 °C and stirred for 8 h. Then water was added and the mixture was extracted with dichloromethane (DCM) three times. The combined organic layer was dried by Na_2_SO_4_, concentrated and purified by column chromatography on silica gel. The products were obtained as red solids.

*Nickel(II)-N-(2-benzyoly-phenyl)-2-piperidino-acetamide/2-amino-3-(1H-indol-3-yl)-3-phenyl-propanoic acid Schiff base complex* **4a**. Yield 57%, m.p. 178–180 °C. ^1^H-NMR (400 MHz, CDCl_3_) δ 8.57–8.48 (m, 2H), 7.70–7.65 (m, 2H), 7.62 (d, *J* = 2.0 Hz, 1H), 7.57–7.49 (m, 3H), 7.37–7.29 (m, 2H), 7.27–7.17 (m, 4H), 7.07–6.98 (m, 2H), 6.84 (t, *J* = 7.5 Hz, 1H), 6.79–6.70 (m, 3H), 4.69 (d, *J* = 2.8 Hz, 1H), 4.38 (d, *J* = 2.8 Hz, 1H), 3.43–3.19 (m, 3H), 3.02 (d, *J* = 13.2 Hz, 1H), 2.34–2.19 (m, 1H), 1.81 (d, *J* = 13.6 Hz, 1H), 1.71–1.57 (m, 2H), 1.54–1.38 (m, 2H), 1.35–1.26 (m, 2H). ^13^C-NMR (100 MHz, CDCl_3_) δ 177.16, 175.72, 170.98, 143.06, 140.40, 135.73, 134.00, 133.72, 132.81, 131.14, 129.72, 129.25, 129.09, 128.85, 127.71, 127.64, 127.10, 127.03, 126.78, 124.29, 123.41, 121.35, 121.09, 118.79, 118.54, 113.66, 111.02, 74.83, 60.17, 55.15, 54.44, 49.66, 22.81, 19.65, 19.32. HRMS (ESI-TOF) calcd. for C_37_H_34_N_4_NaNiO_3_^+^ [M + Na]^+^ 663.1877, found 663.1881.

*Nickel(II)-N-(2-benzyoly-phenyl)-2-piperidino-acetamide/2-amino-3-(1H-indol-3-yl)-3-(3-methoxy-phenyl)-propanoic acid Schiff base complex* **4b**. Yield 62%, m.p. 175–176 °C. ^1^H-NMR (400 MHz, CDCl_3_) δ 8.62–8.53 (m, 2H), 7.60 (d, *J* = 2.4 Hz, 1H), 7.42 (t, *J* = 8.0 Hz, 1H), 7.35–7.29 (m, 2H), 7.29–7.26 (m, 1H), 7.25–7.14 (m, 5H), 7.07–6.98 (m, 3H), 6.90–6.79 (m, 2H), 6.78–6.72 (m, 2H), 4.67 (d, *J* = 2.8 Hz, 1H), 4.37 (d, *J* = 2.8 Hz, 1H), 3.67 (s, 3H), 3.41 (d, *J* = 16.0 Hz, 1H), 3.36–3.26 (m, 2H), 3.05 (d, *J* = 13.2 Hz, 1H), 2.38–2.25 (m, 1H), 1.90 (d, *J* = 13.6 Hz, 1H), 1.77–1.58 (m, 2H), 1.55–1.38 (m, 2H), 1.37–1.28 (m, 2H). ^13^C-NMR (100 MHz, CDCl_3_) δ 177.26, 175.85, 170.97, 160.08, 143.10, 141.93, 135.77, 134.00, 133.69, 132.83, 129.67, 129.65, 129.19, 129.03, 127.69, 127.09, 126.96, 126.88, 124.26, 123.43, 121.42, 121.05, 118.86, 118.63, 115.83, 113.80, 113.55, 111.02, 74.90, 60.14, 55.21, 55.16, 54.40, 49.68, 22.80, 19.65, 19.28. HRMS (ESI-TOF) calcd. for C_38_H_36_N_4_NaNiO_4_^+^ [M + Na]^+^ 693.1982, found 693.1983.

*Nickel(II)-N-(2-benzyoly-phenyl)-2-piperidino-acetamide/2-amino-3-(1H-indol-3-yl)-3-(4-bromo-phenyl)-propanoic acid Schiff base complex* **4c**. Yield 55%, m.p. 185–187 °C. ^1^H-NMR (400 MHz, CDCl_3_) δ 8.64 (brs, 1H), 8.57 (d, *J* = 8.4 Hz, 1H), 7.63 (s, 1H), 7.58 (d, *J* = 8.0 Hz, 2H), 7.50 (d, *J* = 8.0 Hz, 2H), 7.41–7.28 (m, 2H), 7.19 (dd, *J* = 12.8, 7.2 Hz, 4H), 7.04 (t, *J* = 7.2 Hz, 1H), 6.98 (d, *J* = 6.8 Hz, 1H), 6.92–6.82 (m, 2H), 6.81–6.72 (m, 2H), 4.65 (d, *J* = 2.4 Hz, 1H), 4.35 (d, *J* = 2.0 Hz, 1H), 3.49 (d, *J* = 16.4 Hz, 1H), 3.37–3.22 (m, 2H), 3.08 (d, *J* = 13.2 Hz, 1H), 2.33–2.21 (m, 1H), 1.81 (d, *J* = 13.6 Hz, 1H), 1.69–1.48 (m, 2H), 1.47–1.35 (m, 2H), 1.33–1.27 (m, 2H). ^13^C-NMR (100 MHz, CDCl_3_) δ 177.10, 175.91, 171.40, 143.06, 139.46, 135.77, 134.08, 133.56, 132.99, 132.58, 131.80, 129.73, 129.22, 129.07, 127.50, 126.95, 126.88, 126.62, 124.37, 123.50, 121.84, 121.58, 121.20, 119.04, 118.45, 113.04, 111.14, 74.72, 60.05, 55.39, 54.43, 49.14, 22.78, 19.64, 19.25. HRMS (ESI-TOF) calcd. for C_37_H_33_BrN_4_NaNiO_3_^+^ [M + Na]^+^ 741.0982, found 741.0985.

*Nickel(II)-N-(2-benzyoly-phenyl)-2-piperidino-acetamide/2-amino-3-(1H-indol-3-yl)-3-(4-tert-butyl-phenyl)-propanoic acid Schiff base complex* **4d**. Yield 44%, m.p. 173–175 °C. ^1^H–NMR (400 MHz, CDCl_3_) δ 8.57 (d, *J* = 8.4 Hz, 1H), 8.28 (brs, 1H), 7.71 (d, *J* = 2.4 Hz, 1H), 7.65 (d, *J* = 8.0 Hz, 2H), 7.52 (d, *J* = 8.0 Hz, 2H), 7.37–7.29 (m, 2H), 7.24–7.13 (m, 4H), 7.07–7.00 (m, 2H), 6.90–6.82 (m, 2H), 6.78 (d, *J* = 4.0 Hz, 2H), 4.64 (d, *J* = 2.4 Hz, 1H), 4.35 (d, *J* = 2.4 Hz, 1H), 3.42 (d, *J* = 16.0 Hz, 1H), 3.33–3.22 (m, 2H), 3.06 (d, *J* = 13.2 Hz, 1H), 2.30–2.18 (m, 1H), 1.72 (d, *J* = 14.0 Hz, 1H), 1.62–1.44 (m, 2H), 1.35 (s, 9H), 1.31–1.21 (m, 4H). ^13^C-NMR (100 MHz, CDCl_3_) δ 177.20, 175.71, 170.83, 150.13, 143.06, 137.29, 135.58, 133.94, 133.72, 132.75, 130.67, 129.61, 129.19, 129.00, 127.59, 127.08, 127.02, 126.77, 125.75, 124.10, 123.46, 121.38, 121.10, 118.77, 118.53, 114.48, 110.88, 75.30, 59.93, 54.90, 54.17, 49.29, 31.59, 22.83, 19.65, 19.16. HRMS (ESI-TOF) calcd. for C_41_H_42_N_4_NaNiO_3_^+^ [M + Na]^+^ 719.2503, found 719.2505.

*Nickel(II)-N-(2-benzyoly-phenyl)-2-piperidino-acetamide/2-amino-3-(1H-indol-3-yl)-3-(3-chloro-phenyl)-propanoic acid Schiff base complex* **4e**. Yield 58%, m.p. 192–193 °C. ^1^H-NMR (400 MHz, CDCl_3_) δ 8.61 (d, *J* = 8.4 Hz, 1H), 8.55 (brs, 1H), 7.85 (s, 1H), 7.62 (d, *J* = 2.0 Hz, 1H), 7.50–7.43 (m, 1H), 7.42–7.30 (m, 4H), 7.25–7.18 (m, 3H), 7.14 (t, *J* = 7.6 Hz, 1H), 7.05 (t, *J* = 7.6 Hz, 1H), 6.99 (d, *J* = 7.6 Hz, 1H), 6.93–6.82 (m, 2H), 6.80–6.74 (m, 2H), 4.68 (d, *J* = 2.4 Hz, 1H), 4.40 (d, *J* = 2.4 Hz, 1H), 3.46 (d, *J* = 16.4Hz, 1H), 3.38–3.25 (m, 2H), 3.08 (d, *J* = 13.2 Hz, 1H), 2.40–2.28 (m, 1H), 1.87 (d, *J* = 13.6 Hz, 1H), 1.72–1.59 (m, 2H), 1.56–1.27 (m, 4H). ^13^C-NMR (100 MHz, CDCl_3_) δ 177.02, 175.97, 171.38, 143.16, 142.62, 135.69, 134.68, 134.06, 133.58, 133.00, 130.73, 130.05, 129.70, 129.15, 129.07, 129.04, 127.79, 127.56, 126.95, 126.78, 126.66, 124.44, 123.45, 121.63, 121.11, 119.07, 118.36, 112.94, 111.09, 74.75, 60.21, 55.43, 54.39, 49.06, 22.75, 19.66, 19.23. HRMS (ESI-TOF) calcd. for C_37_H_33_ClN_4_NaNiO_3_^+^ [M + Na]^+^ 697.1487, found 697.1489.

*Nickel(II)-N-(2-benzyoly-phenyl)-2-piperidino-acetamide/2-amino-3-(1H-indol-3-yl)-3-(4-trifluoromethyl-phenyl)-propanoic acid Schiff base complex* **4f**. Yield 60%, m.p. 201–202 °C. ^1^H-NMR (400 MHz, CDCl_3_) δ 8.73 (brs, 1H), 8.59 (d, *J* = 8.4 Hz, 1H), 7.77–7.65 (m, 5H), 7.38–7.31 (m, 1H), 7.23–7.18 (m, 2H), 7.17–7.10 (m, 3H), 7.08–7.02 (m, 1H), 6.99–6.94 (m, 1H), 6.93–6.87 (m, 2H), 6.82–6.74 (m, 2H), 4.69 (d, *J* = 2.4 Hz, 1H), 4.47 (d, *J* = 2.0 Hz, 1H), 3.46 (d, *J* = 16.4 Hz, 1H), 3.27 (t, *J* = 12.0 Hz, 1H), 3.17 (d, *J* = 16.4 Hz, 1H), 3.07 (d, *J* = 13.2 Hz, 1H), 2.20 (t, *J* = 12.0 Hz, 1H), 1.69–1.48 (m, 3H), 1.44–1.20 (m, 4H). ^13^C-NMR (100 MHz, CDCl_3_) δ 177.05, 175.88, 171.65, 144.76, 143.09, 135.74, 134.12, 133.48, 133.08, 131.12, 130.02, 129.72, 129.17, 129.05, 127.42, 126.87, 126.83, 126.57, 125.64, 125.60, 125.56, 125.53, 124.51, 123.54, 122.94, 121.69, 121.26, 119.17, 118.25, 112.93, 111.19, 74.91, 59.88, 55.36, 54.33, 49.41, 22.70, 19.28, 19.14. HRMS (ESI-TOF) calcd. for C_38_H_33_F_3_N_4_NaNiO_3_^+^ [M + Na]^+^ 731.1750, found 731.1753.

*Nickel(II)-N-(2-benzyoly-phenyl)-2-piperidino-acetamide/2-amino-3-(1H-indol-3-yl)-3-p-tolyl-propanoic acid Schiff base complex* **4g**. Yield 66%, m.p. 171–173 °C. ^1^H-NMR (400 MHz, CDCl_3_) δ 8.54 (d, *J* = 8.4 Hz, 1H), 8.45 (brs, 1H), 7.63–7.53 (m, 3H), 7.36–7.30 (m, 4H), 7.25–7.17 (m, 4H), 7.02 (dd, *J* = 14.0, 7.6 Hz, 2H), 6.84 (t, *J* = 7.6 Hz, 1H), 6.79–6.73 (m, 3H), 4.66 (d, *J* = 2.8 Hz, 1H), 4.34 (d, *J* = 2.8 Hz, 1H), 3.42 (d, *J* = 16.0 Hz, 1H), 3.33–3.19 (m, 2H), 3.02 (d, *J* = 13.2 Hz, 1H), 2.44 (s, 3H), 2.32–2.21 (m, 1H), 1.79 (d, *J* = 13.6 Hz, 1H), 1.67–1.56 (m, 1H), 1.55–1.23 (m, 5H). ^13^C-NMR (100 MHz, CDCl_3_) δ 177.18, 175.70, 170.86, 143.02, 137.36, 137.17, 135.75, 133.96, 133.73, 132.76, 130.98, 129.67, 129.48, 129.22, 129.05, 127.66, 127.10, 127.07, 126.79, 124.16, 123.45, 121.32, 121.09, 118.76, 118.61, 113.89, 110.98, 74.97, 60.08, 55.12, 54.35, 49.41, 22.80, 21.18, 19.59, 19.27. HRMS (ESI-TOF) calcd. for C_38_H_36_N_4_NaNiO_3_^+^ [M + Na]^+^ 677.2033, found 677.2035.

*Nickel(II)-N-(2-benzyoly-phenyl)-2-piperidino-acetamide/2-amino-3-(1H-indol-3-yl)-3-(4-nitro-phenyl)-propanoic acid Schiff base complex* **4h**. Yield 60%, m.p. 184–186 °C. ^1^H-NMR (400 MHz, CDCl_3_) δ 8.59 (d, *J* = 8.8 Hz, 1H), 8.42 (brs, 1H), 8.27 (d, *J* = 8.8 Hz, 2H), 7.82 (d, *J* = 8.4 Hz, 2H), 7.65 (d, *J* = 2.0 Hz, 1H), 7.38–7.32 (m, 2H), 7.28 (d, *J* = 8.4 Hz, 1H), 7.25–7.18 (m, 2H), 7.13–7.04 (m, 2H), 6.97–6.87 (m, 3H), 6.82–6.74 (m, 2H), 4.75 (d, *J* = 2.4 Hz, 1H), 4.60 (d, *J* = 2.0 Hz, 1H), 3.46 (d, *J* = 16.4 Hz, 1H), 3.29 (t, *J* = 11.6 Hz, 1H), 3.14 (d, *J* = 16.4 Hz, 1H), 3.07 (d, *J* = 13.2 Hz, 1H), 2.24 (t, *J* = 11.6 Hz, 1H), 1.56–1.46 (m, 2H), 1.45–1.26 (m, 5H). ^13^C-NMR (100 MHz, CDCl_3_) δ 176.83, 175.79, 171.94, 148.14, 147.66, 143.12, 135.74, 134.19, 133.50, 133.26, 131.32, 129.75, 129.13, 129.09, 127.48, 126.87, 126.64, 126.55, 124.62, 123.60, 123.54, 122.09, 121.31, 119.57, 118.31, 112.51, 111.18, 74.81, 60.24, 55.72, 54.48, 49.35, 22.67, 19.60, 19.24. HRMS (ESI-TOF) calcd. for C_37_H_33_N_5_NaNiO_5_^+^ [M + Na]^+^ 708.1727, found 708.1733.

*Nickel(II)-N-(2-benzyoly-phenyl)-2-piperidino-acetamide/2-amino-3-(1H-indol-3-yl)-3-ferrocene-propanoic acid Schiff base complex* **4i**. Yield 43%, m.p. 211–213 °C. ^1^H-NMR (400 MHz, CDCl_3_) δ 8.74 (brs, 1H), 8.52 (d, *J* = 8.0 Hz, 1H), 7.53 (d, *J* = 2.4 Hz, 1H), 7.47–7.42 (m, 2H), 7.39–7.35 (m, 1H), 7.34–7.27 (m, 2H), 7.18 (d, *J* = 7.6 Hz, 1H), 7.13–7.07 (m, 1H), 7.01–6.92 (m, 3H), 6.78–6.66 (m, 2H), 4.51 (d, *J* = 2.4 Hz, 2H), 4.43 (s, 1H), 4.36 (d, *J* = 3.6 Hz, 1H), 4.33 (s, 2H), 3.72 (s, 5H), 3.38 (s, 2H), 3.27 (td, *J* = 12.4, 2.8 Hz, 1H), 3.01 (d, *J* = 13.2 Hz, 1H), 2.37–2.24 (m, 1H), 1.95 (d, *J* = 13.6 Hz, 1H), 1.70–1.63 (m, 1H), 1.54–1.46 (m, 1H), 1.44–1.36 (m, 1H), 1.34–1.25 (m, 3H). ^13^C-NMR (100 MHz, CDCl_3_) δ 176.43, 174.67, 169.60, 141.86, 133.99, 133.16, 132.73, 131.60, 128.54, 128.22, 128.10, 127.92, 126.68, 126.49, 126.20, 124.06, 122.16, 120.66, 119.92, 118.20, 112.29, 110.01, 87.47, 75.00, 70.52, 69.20, 67.74, 67.43, 66.91, 59.08, 54.12, 53.30, 41.88, 21.83, 18.66, 18.19. HRMS (ESI-TOF) calcd. for C_41_H_38_FeN_4_NaNiO_3_^+^ [M + Na]^+^ 771.1539, found 771.1546.

*Nickel(II)-N-(2-benzyoly-phenyl)-2-piperidino-acetamide/2-amino-3-(2-methyl-1H-indol-3-yl)-3-phenyl-propanoic acid Schiff base complex* **4j**. Yield 45%, m.p. 177–178 °C. ^1^H-NMR (400 MHz, CDCl_3_) δ 9.19 (brs, 1H), 8.49 (dj, *J* = 8.4 Hz, 1H), 7.77–7.54 (m, 2H), 7.53–7.44 (m, 2H), 7.43–7.35 (m, 2H), 7.34–7.28 (m, 1H), 7.18–7.09 (m, 2H), 7.08–6.95 (m, 6H), 6.88–6.82 (m, 1H), 6.82–6.75 (m, 1H), 4.93 (d, *J* = 3.2 Hz, 1H), 4.43 (brs, 1H), 3.27–3.09 (m, 2H), 2.89 (d, *J* = 12.4 Hz, 1H), 2.23–1.87 (m, 4H), 1.84–1.64 (m, 1H), 1.61–1.46 (m, 1H), 1.43–1.20 (m, 3H), 1.18–0.87 (m, 3H). ^13^C-NMR (100 MHz, CDCl_3_) δ 177.66, 175.80, 170.08, 142.99, 136.02, 133.78, 133.68, 132.72, 129.96, 129.48, 129.43, 127.88, 127.79, 127.29, 126.92, 126.10, 123.51, 121.27, 121.13, 119.84, 110.25, 73.50, 59.63, 54.38, 48.35, 22.66, 19.43, 19.19, 12.64. HRMS (ESI-TOF) calcd. for C_38_H_36_N_4_NaNiO_3_^+^ [M + Na]^+^ 677.2033, found 677.2037.

*Nickel(II)-N-(2-benzyoly-phenyl)-2-piperidino-acetamide/2-amino-3-(4-bromo-1H-indol-3-yl)-3-phenyl-propanoic acid Schiff base complex* **4k**. Yield 43%, m.p.172–173 °C. ^1^H-NMR (400 MHz, CDCl_3_) δ 8.77 (brs, 1H), 8.60 (d, *J* = 8.4 Hz, 1H), 8.36 (d, *J* = 2.4 Hz, 1H), 7.76 (d, *J* = 5.6 Hz, 2H), 7.49–7.40 (m, 3H), 7.38–7.33 (m, 2H), 7.18–7.01 (m, 6H), 6.88–6.76 (m, 3H), 5.11 (d, *J* = 1.2 Hz, 1H), 4.53 (d, *J* = 1.6 Hz, 1H), 3.53–3.43 (m, 2H), 3.32 (td, *J* = 12.8, 3.2 Hz, 1H), 3.10 (d, *J* = 13.2 Hz, 1H), 2.21–2.10 (m, 1H), 1.62–1.13 (m, 7H). ^13^C-NMR (100 MHz, CDCl_3_) δ 177.90, 175.98, 171.39, 143.07, 140.51, 137.05, 134.09, 133.60, 132.83, 131.64, 129.49, 129.04, 128.65, 128.50, 127.91, 127.18, 126.99, 126.58, 126.48, 124.00, 123.85, 123.51, 122.15, 121.17, 115.59, 113.30, 110.51, 76.49, 60.21, 54.90, 54.36, 49.21, 22.83, 19.58, 19.25. HRMS (ESI-TOF) calcd. for C_37_H_33_BrN_4_NaNiO_3_^+^ [M + Na]^+^ 741.0982, found 741.0986.

*Nickel(II)-N-(2-benzyoly-phenyl)-2-piperidino-acetamide/2-amino-3-(5-chloro-1H-indol-3-yl)-3-phenyl-propanoic acid Schiff base complex* **4l**. Yield 53%, m.p. 173–175 °C. ^1^H-NMR (400 MHz, CDCl_3_) δ 8.79 (brs, 1H), 8.51 (d, *J* = 8.4 Hz, 1H), 7.72–7.65 (m, 2H), 7.62–7.54 (m, 4H), 7.44–7.27 (m, 5H), 7.17–7.12 (m, 1H), 7.04 (d, *J* = 8.4 Hz, 1H), 6.91 (dd, *J* = 8.4, 1.7 Hz, 1H), 6.82–6.74 (m, 2H), 6.57 (d, *J* = 1.2 Hz, 1H), 4.65 (d, *J* = 3.2 Hz, 1H), 4.29 (d, *J* = 3.2 Hz, 1H), 3.41–3.18 (m, 3H), 3.01 (d, *J* = 13.2 Hz, 1H), 2.39–2.27 (m, 1H), 1.70–1.58 (m, 2H), 1.55–1.43 (m, 2H), 1.39–1.30 (m, 2H). ^13^C-NMR (100 MHz, CDCl_3_) δ 176.14, 174.53, 170.04, 141.99, 138.69, 133.01, 132.95, 132.64, 131.87, 130.08, 129.23, 128.32, 128.31, 127.95, 126.92, 126.82, 126.58, 126.02, 125.96, 124.77, 123.46, 122.38, 120.60, 120.10, 116.58, 112.09, 111.08, 73.38, 59.14, 54.26, 53.54, 47.93, 21.78, 18.68, 18.35. HRMS (ESI-TOF) calcd. for C_37_H_33_ClN_4_NaNiO_3_^+^ [M + Na]^+^ 697.1487, found 697.1491.

*Nickel(II)-N-(2-benzyoly-phenyl)-2-piperidino-acetamide/2-amino-3-(6-fluoro-1H-indol-3-yl)-3-phenyl-propanoic acid Schiff base complex* **4m**. Yield 57%, m.p. 181–182 °C. ^1^H-NMR (400 MHz, CDCl_3_) δ 9.01 (brs, 1H), 8.52 (d, *J* = 8.4 Hz, 1H), 7.67–7.59 (m, 2H), 7.57–7.49 (m, 3H), 7.43–7.36 (m, 2H), 7.35–7.26 (m, 4H), 7.07 (d, *J* = 7.6 Hz, 1H), 6.85 (dd, *J* = 9.6, 2.0 Hz, 1H), 6.81–6.74 (m, 2H), 6.56 (td, *J* = 9.6, 2.0 Hz, 1H), 6.47–6.39 (m, 1H), 4.69 (d, *J* = 3.2 Hz, 1H), 4.32 (d, *J* = 3.2 Hz, 1H), 3.43–3.18 (m, 3H), 3.01 (d, *J* = 13.2 Hz, 1H), 2.36–2.25 (m, 1H), 1.89 (d, *J* = 13.6 Hz, 1H), 1.82–1.57 (m, 2H), 1.55–1.29 (m, 5H). ^13^C-NMR (100 MHz, CDCl_3_) δ 176.18, 174.59, 169.97, 141.97, 138.96, 134.84, 134.71, 132.96, 132.72, 131.85, 130.10, 128.80, 128.28, 128.21, 127.88, 126.81, 126.18, 125.96, 123.67, 123.63, 122.36, 122.16, 120.10, 117.96, 117.86, 112.15, 106.33, 106.09, 96.51, 96.26, 73.55, 59.13, 54.26, 53.53, 48.51, 21.77, 18.60, 18.34. HRMS (ESI-TOF) calcd. for C_37_H_33_FN_4_NaNiO_3_^+^ [M + Na]^+^ 681.1782, found 681.1786.

*Nickel(II)-N-(2-benzyoly-phenyl)-2-piperidino-acetamide/2-amino-3-(7-methyl-1H-indol-3-yl)-3-phenyl-propanoic acid Schiff base complex* **4n**. Yield 55%, m.p. 179–181 °C. ^1^H-NMR (400 MHz, CDCl_3_) δ 8.53 (d, *J* = 8.4 Hz, 1H), 8.09–8.04 (m, 1H), 7.74–7.67 (m, 3H), 7.62–7.49 (m, 3H), 7.41 (td, *J* = 7.6, 1.2 Hz, 1H), 7.36–7.21 (m, 4H), 7.08 (d, *J* = 7.6 Hz, 1H), 6.84 (d, *J* = 7.2 Hz, 1H), 6.80–6.73 (m, 3H), 6.60 (d, *J* = 8.0 Hz, 1H), 4.69 (d, *J* = 2.8 Hz, 1H), 4.36 (d, *J* = 2.8 Hz, 1H), 3.42–3.18 (m, 3H), 3.02 (d, *J* = 13.2 Hz, 1H), 2.38 (s, 3H), 2.31–2.21 (m, 1H), 1.81 (d, *J* = 13.6 Hz, 1H), 1.71–1.61 (m, 2H), 1.55–1.28 (m, 4H). ^13^C-NMR (100 MHz, CDCl_3_) δ 177.03, 175.70, 170.92, 143.07, 140.52, 135.22, 133.97, 133.78, 132.79, 131.19, 129.76, 129.31, 129.08, 128.84, 127.70, 127.64, 127.11, 127.01, 126.34, 123.87, 123.41, 122.07, 121.06, 120.00, 119.10, 116.36, 114.43, 74.76, 60.18, 55.14, 54.42, 49.81, 22.81, 19.64, 19.32, 16.54. HRMS (ESI-TOF) calcd. for C_38_H_36_N_4_NaNiO_3_^+^ [M + Na]^+^ 677.2033, found 677.2038.

### 3.4. General Procedure for the Synthesis of N-Fmoc-β-Substituted-Tryptophan ***5a***

3 mol/L HCl (1 mL) was added to a solution of the complex **4a** (0.2 mmol) dissolved in THF (4 mL). The red color of the solution disappeared immediately for a transient moment then became darker. Three minutes later, the reaction was concentrated under vacuum to half of the original volume. Additional water (2 mL) was added and the residue was extracted with ethyl acetate until the color of the aqueous layer almost disappeared. Then, the aqueous portion was transferred to a clean flask, and solid NaHCO3 (0.8 mmol) was carefully added with stirring to neutralize the solution, followed by Na2EDTA (0.2 mmol), and was stirred for 5 min. Additional solid NaHCO3 (0.8 mmol) was added, followed by a solution of Fmoc-OSu (0.2 mmol) in acetonitrile (3 mL). The reaction was stirred for 24 h under room temperature, concentrated in vacuum, adjusted to pH = 3 with 10% citric acid, and extracted with ethyl acetate three times. The combined organic layer was dried, concentrated, and purified by column chromatography on silica to give compound **5a** as a white solid.

*2-((((9H-fluoren-9-yl)methoxy)carbonyl)amino)-3-(1H-indol-3-yl)-3-phenylpropanoic acid* **5a**. ^1^H-NMR (400 MHz, DMSO) δ 10.97 (s, 1H), 9.52 (brs, 1H), 8.12–7.78 (m, 2H), 7.57 (d, *J* = 7.2 Hz, 2H), 7.46–7.30 (m, 5H), 7.29–7.09 (m, 6H), 7.02 (t, *J* = 7.2 Hz, 1H), 6.94–6.78 (m, 2H), 5.40 (d, *J* = 7.2 Hz, 1H), 5.09 (d, *J* = 4.0 Hz, 1H), 4.41 (d, *J* = 4.0 Hz, 1H), 3.00 (d, *J* = 16.0 Hz, 1H), 2.62 (d, *J* = 16.0 Hz, 1H). ESI-MS (*m*/*z*): calcd. for [M − H]^−^ 501.2, found 501.4.

## 4. Conclusions

The base-promoted three-component reaction of aldehydes, indoles, and the nickel(II) complex as an equivalent of glycine was reported for the first time. A series of modified nickel(II) complexes, which could be decomposed via a simple procedure to afford β-substituted-tryptophans, were obtained through this reaction. Despite the moderate yield, the reaction worked smoothly under mild conditions and the procedure was easy to handle. The relative configuration of the product was determined by X-ray crystallography. Further studies could be focused on the asymmetric reaction of these three components and introduction of a wider range of substrates, such as aliphatic aldehydes and other heterocycles except indole, to enhance the diversity of this reaction.

## Figures and Tables

**Table 1:**
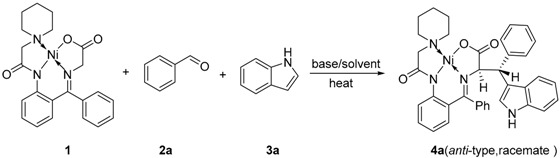
Optimization of the reaction conditions.

Entry	Base	Solvent	Temp (°C)	Yield (%) ^a^
1	NaOH	MeCN	80	NR ^b^
2	NaOH	EtOH	80	15
3	NaOH	Glycerol	80	28
5	LiOH	Glycerol	80	32
6	DIEA	Glycerol	80	NR
7	DBU	Glycerol	80	11
8	TMG	Glycerol	80	46
9	TMG	Glycerol	40	NR
10	TMG	Glycerol	70	57
11	TMG	Glycerol	90	45

The reactions were all conducted for 8 h and bases were used in 3 equiv.; ^a^ Isolated yield for the single diastereoisomer; ^b^ No reaction.

**Table 2:**
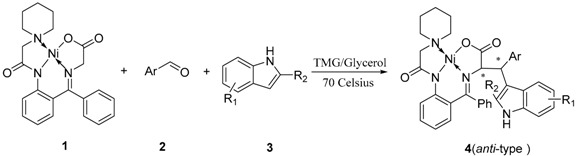
Three-component condensation of nickel(II) glycinate, aromatic aldehydes, and indoles.

Entry	Compound	Ar	R1	R2	Yield(%) ^a^
1	**4a**	Phenyl	H	H	57
2	**4b**	3-OMe phenyl	H	H	62
3	**4c**	4-Br phenyl	H	H	55
4	**4d**	4-tBu phenyl	H	H	44
5	**4e**	3-Cl phenyl	H	H	58
6	**4f**	4-CF3 phenyl	H	H	60
7	**4g**	4-Me phenyl	H	H	66
8	**4h**	4-NO2 phenyl	H	H	35
9	**4i**	Ferrocene	H	H	43
10	**4j**	Phenyl	H	Me	45
11	**4k**	Phenyl	4-Br	H	43
12	**4l**	Phenyl	5-Cl	H	53
13	**4m**	Phenyl	6-F	H	57
14	**4n**	Phenyl	7-Me	H	55
15	**4o**	4-(dimethylamino) phenyl	H	H	NR ^b^
16	**4p**	4-Pyridine	H	H	NP ^c^

^a^ Isolated yield for the single diastereoisomer; ^b^ Not reaction; ^c^ No target product.

## Supplementary Materials

The following are available online, Figure S1: Crystal structure of **4a**, Figure S2: Structures and NOE spectra of compound **4a** and **4j**, Table S1: Crystal data and structure refinement for **4a**.
