# Supplementary material for: Base-Promoted Synthesis of β-Substituted-Tryptophans via a Simple and Convenient Three-Component Condensation of Nickel(II) Glycinate
Source: Molecules. 2017 Apr 27;22(5):695. doi: 10.3390/molecules22050695 (PMC6154671; doi:10.3390/molecules22050695)

Supplementary Materials

Table S1. Crystal data and structure refinement for **4a**

Empirical formula	$C_{38}H_{35}Cl_3N_4NiO_3$
Formula weight	760.74
Temperature/K	293.15
Crystal system	triclinic
Space group	P-1
a/Å	9.3484(3)
b/Å	11.3583(4)
c/Å	17.0360(6)
α/°	87.8580(10)
β/°	76.1330(10)
γ/°	79.8190(10)
Volume/Å^3	1728.52(10)
Z	2
ρ_{calc}g/cm^3	1.462
μ/mm^{-1}	3.300
F(000)	788.0
Radiation	CuKα (λ = 1.54184)
2Θ range for data collection/°	10.698 to 136.618
Index ranges	$-11 \le h \le 11$, $-13 \le k \le 13$, $-20 \le l \le 18$
Reflections collected	47661
Independent reflections	6260 [R_{int} = 0.0255, R_{sigma} = 0.0160]
Data/restraints/parameters	6260/0/452
Goodness-of-fit on F^2	1.039
Final R indexes [I>=2σ (I)]	R_1 = 0.0483, wR_2 = 0.1221
Final R indexes [all data]	R_1 = 0.0492, wR_2 = 0.1228
Largest diff. peak/hole / e Å^{-3}	0.86/-0.88

Figure S1 Crystal structure of **4a**

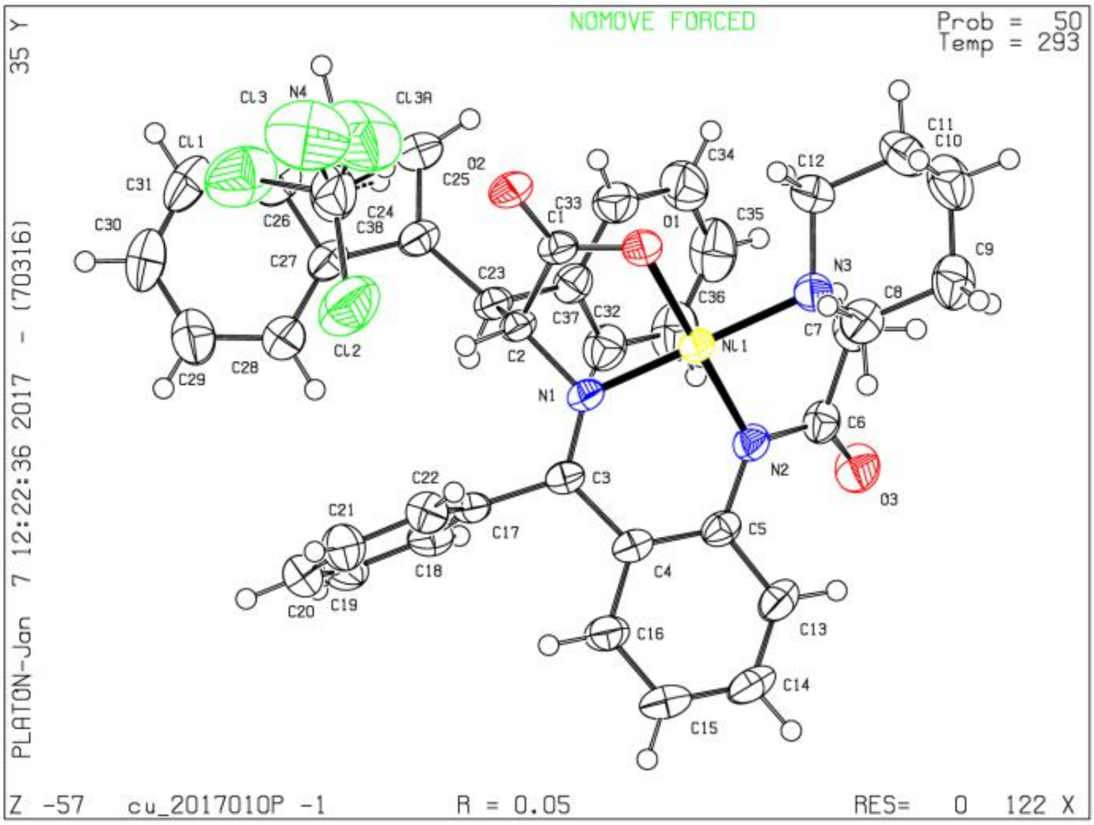

Figure S2 Structures and NOE spectra of compound **4a** and **4j**

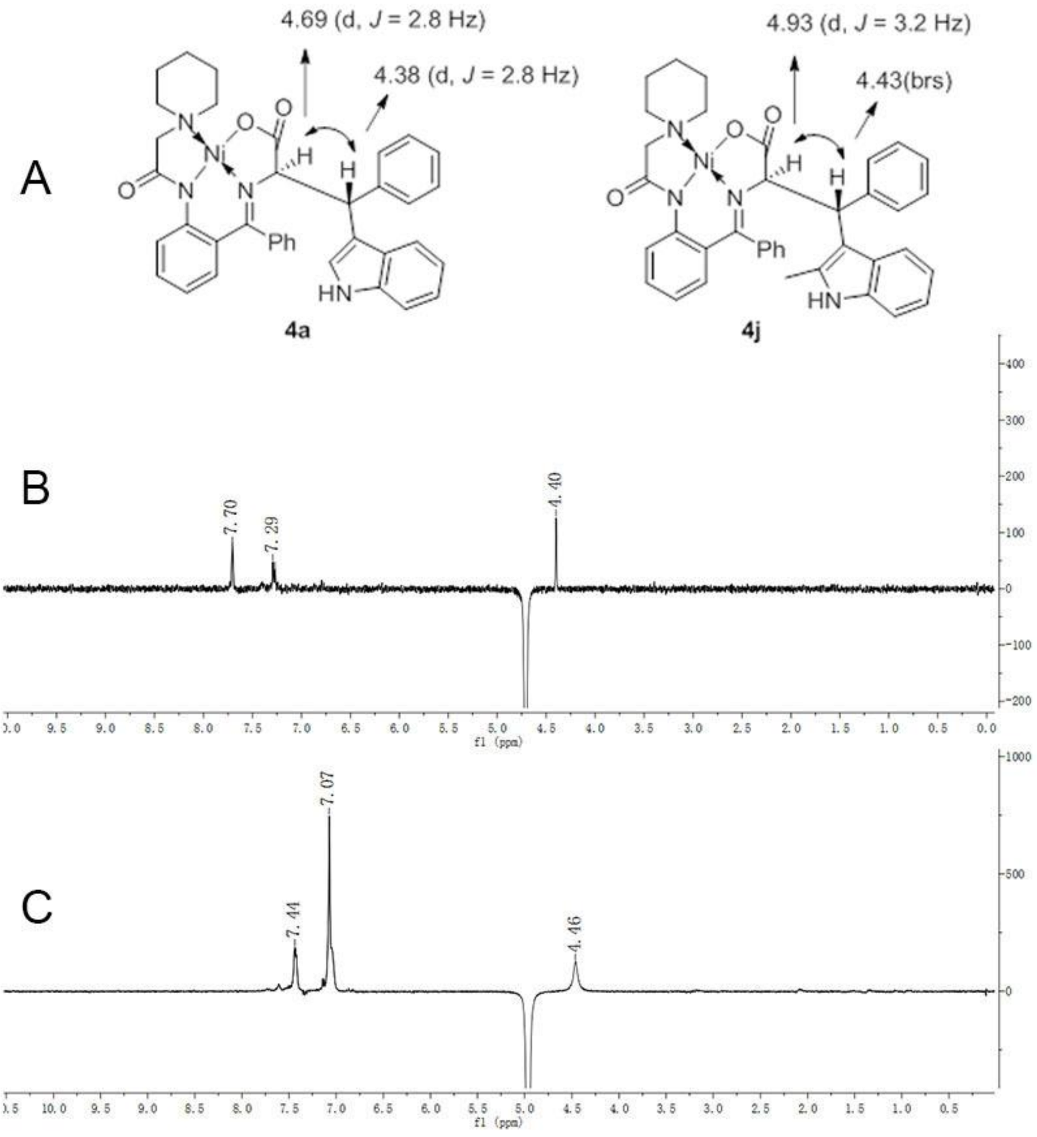

(A)Structures and related 1HNMR data of compound 4a and 4j. NOE spectra of 4a (B) and 4j (C) both show that the protons on the two chiral carbon**s** correlate with each other. NOE spectrum of 4j (C) indicates that the proton at 4.93 is not correlated with the methyl group in space.

Copies of NMR spectra

^{1}HNMR spectrum of compound **4a**

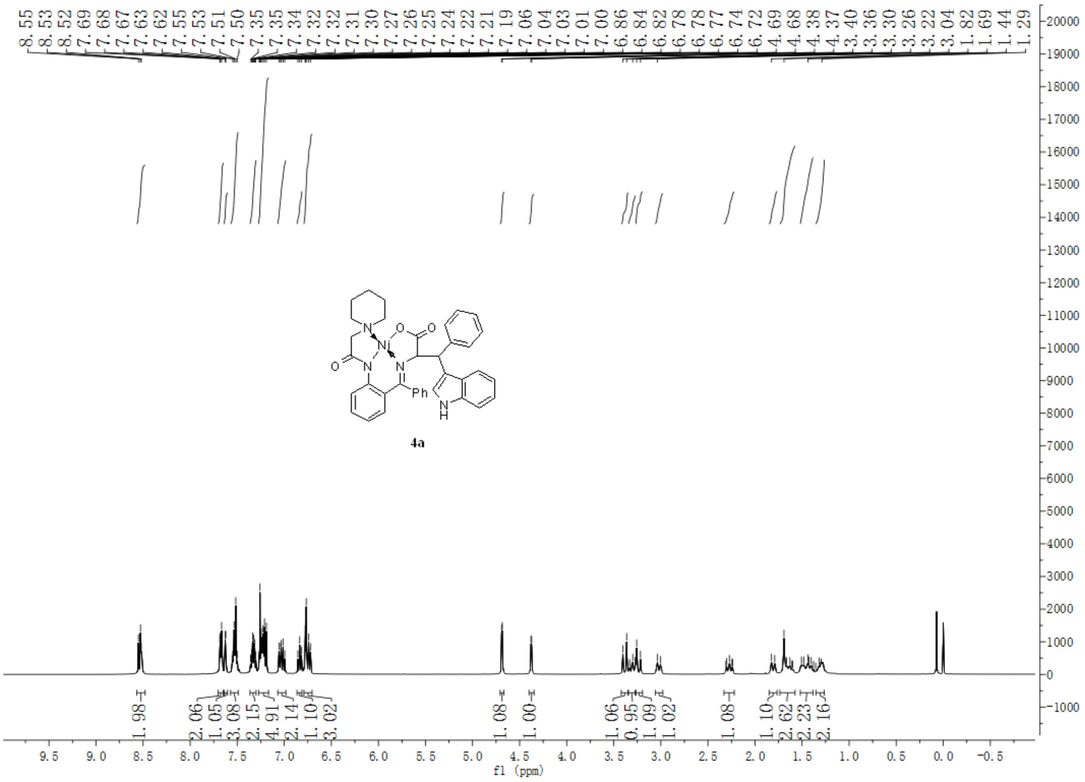

^{13}CNMR spectrum of compound **4a**

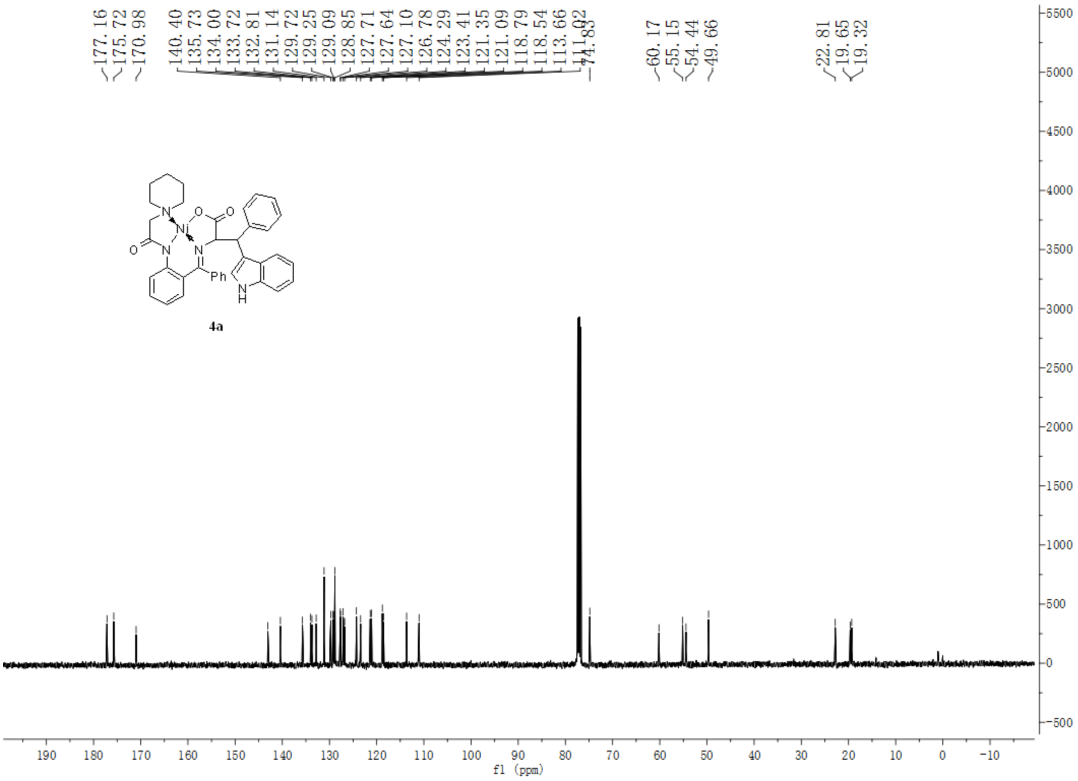

^{1}HNMR spectrum of compound **4b**

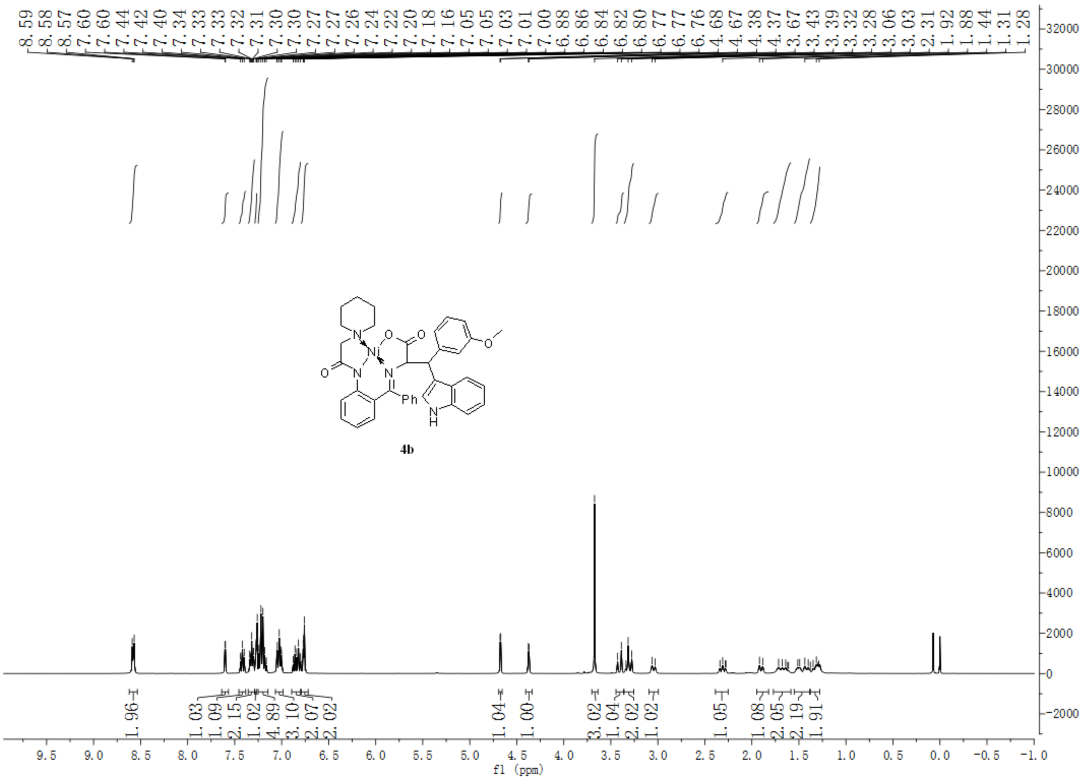

^{13}CNMR spectrum of compound **4b**

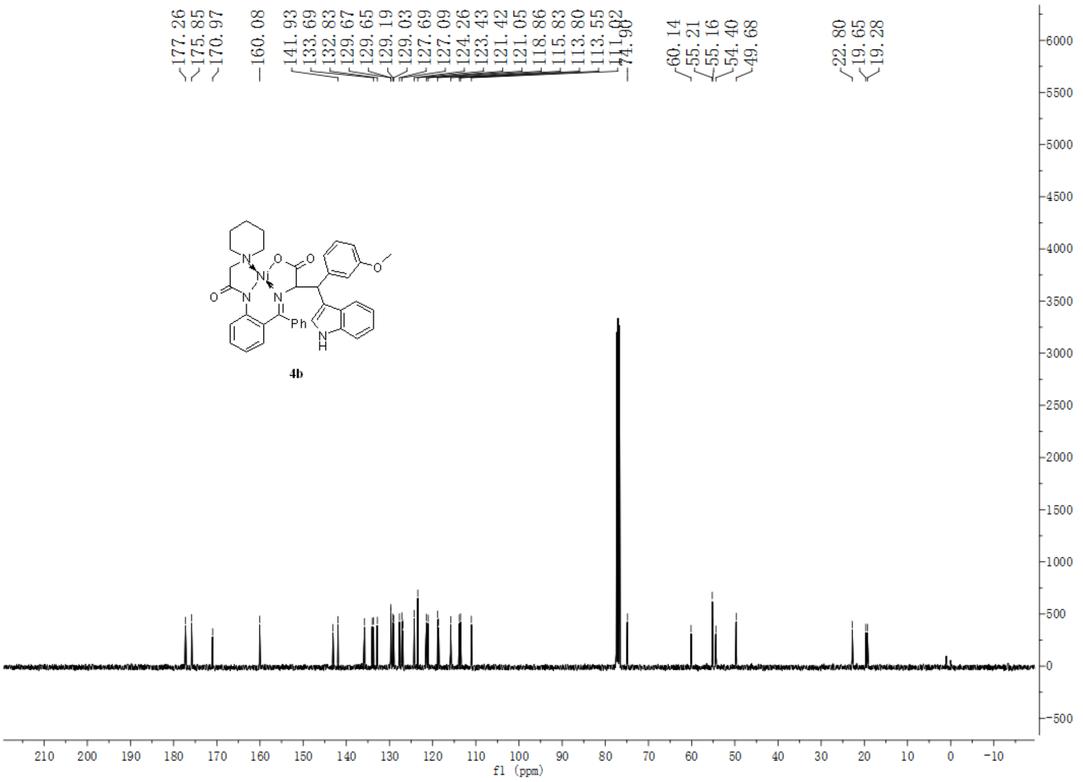

^{1}HNMR spectrum of compound **4c**

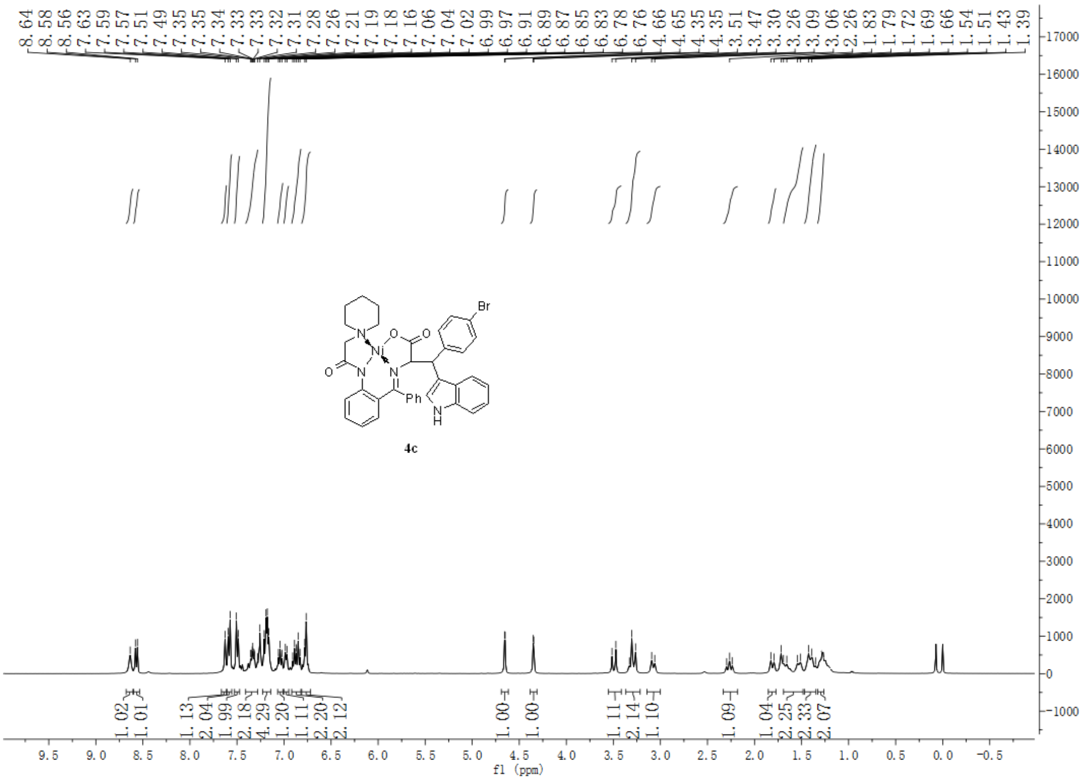

^{13}CNMR spectrum of compound **4c**

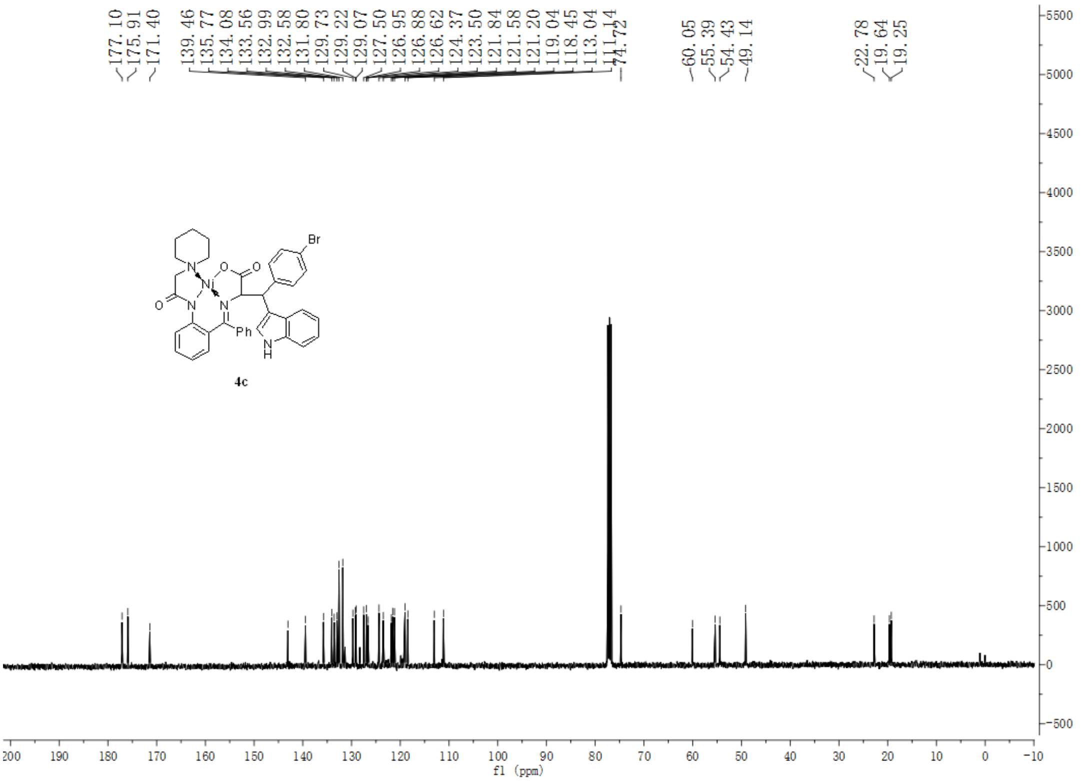

^{1}HNMR spectrum of compound **4d**

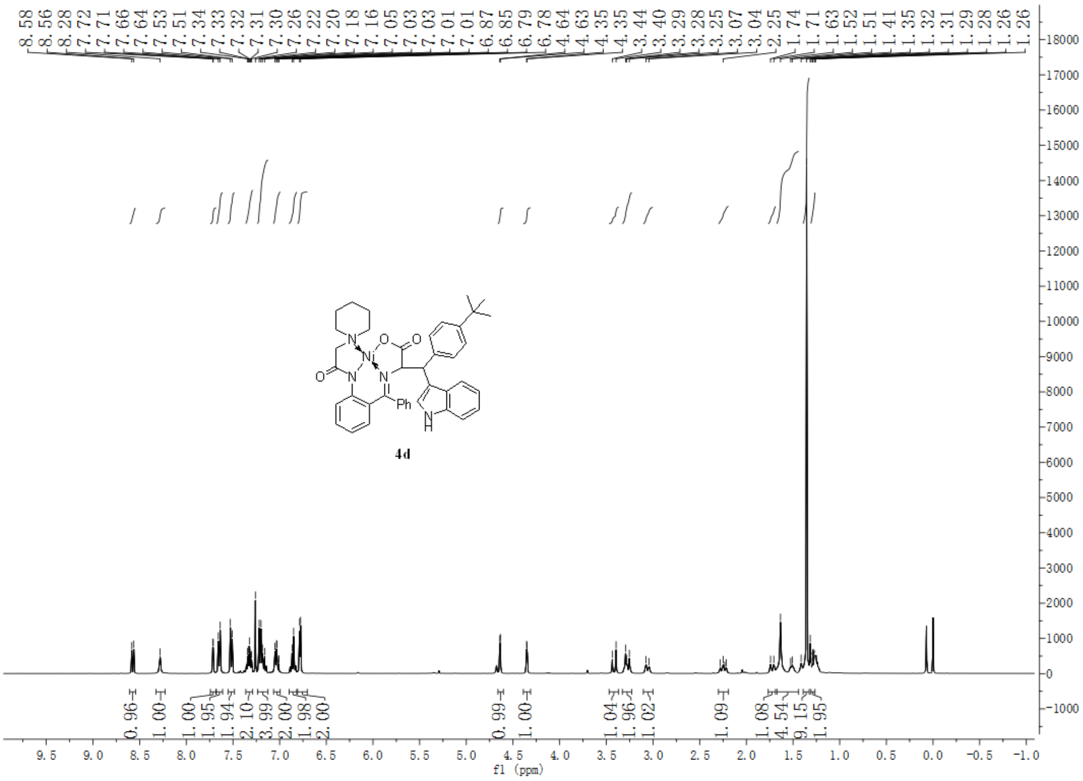

^{13}CNMR spectrum of compound **4d**

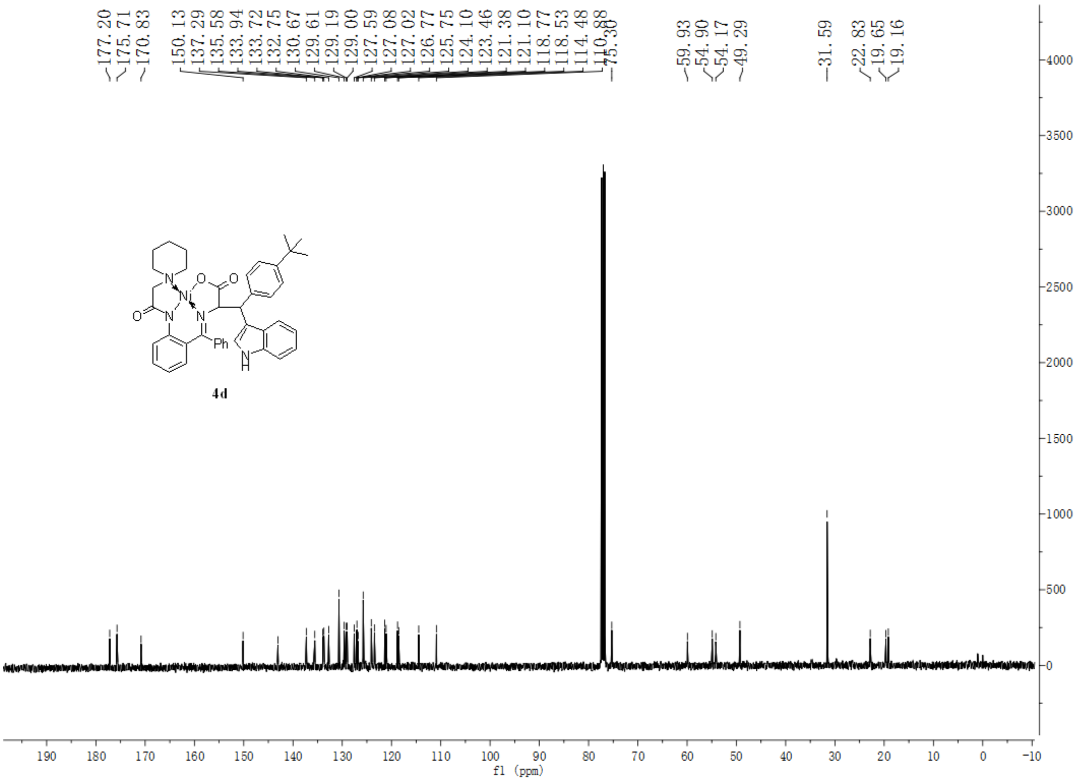

^{1}HNMR spectrum of compound **4e**

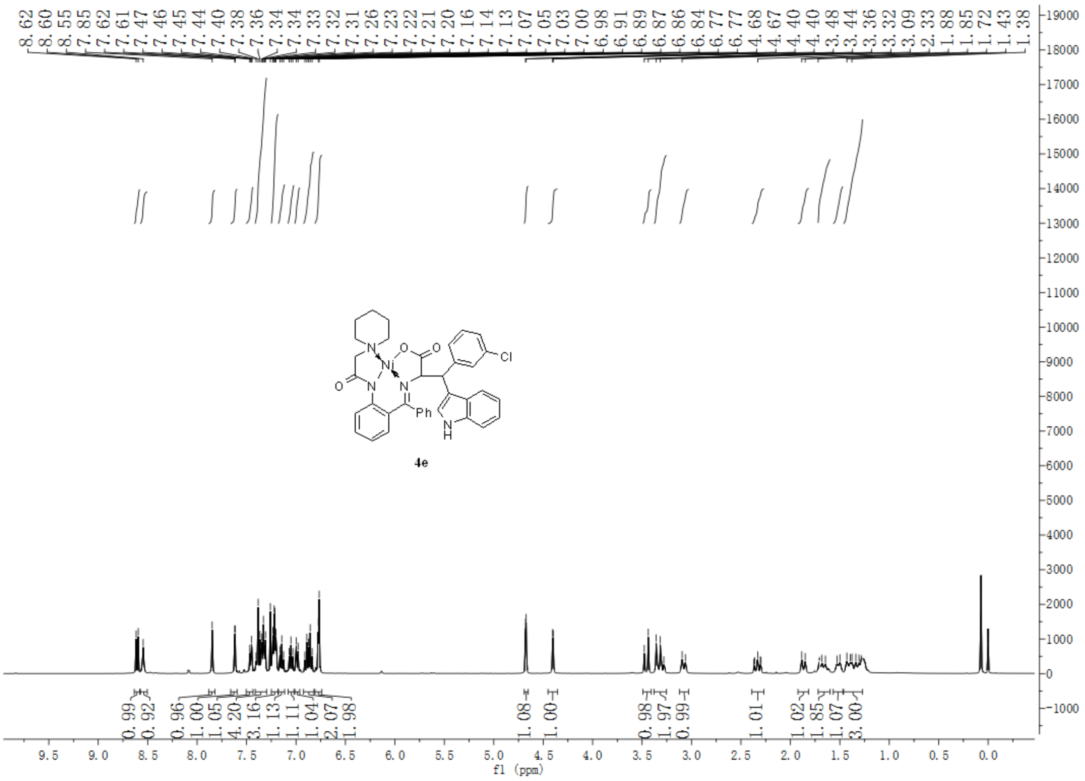

^{13}CNMR spectrum of compound **4e**

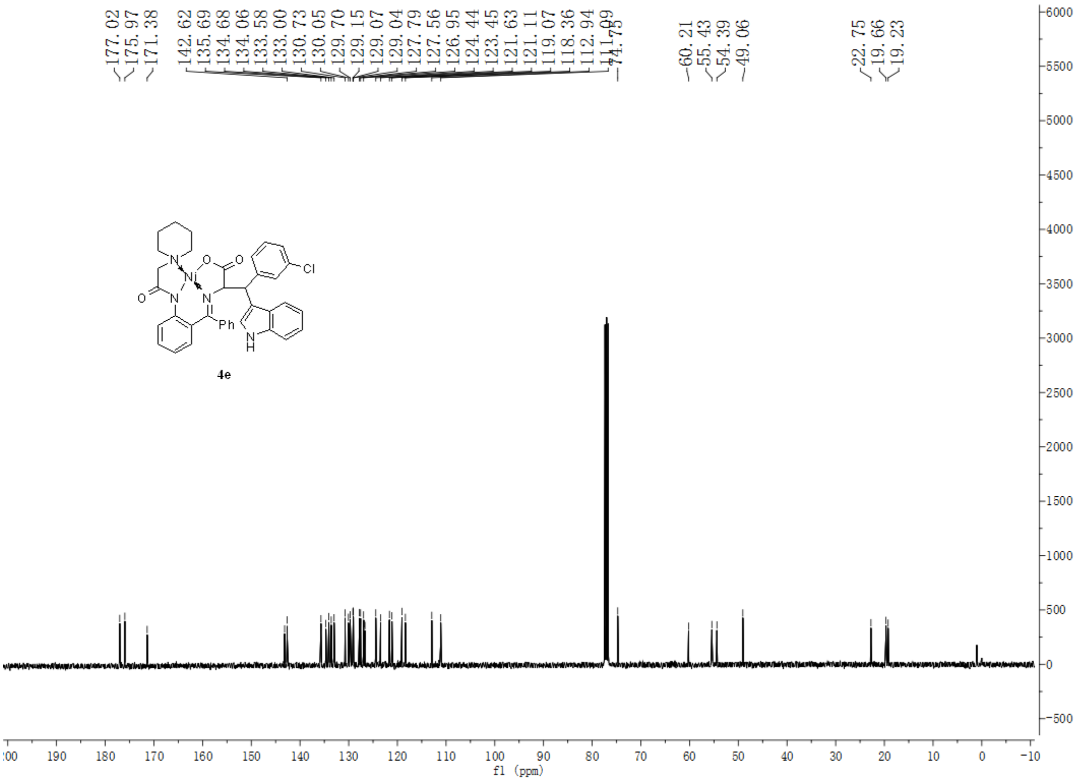

^{1}HNMR spectrum of compound **4f**

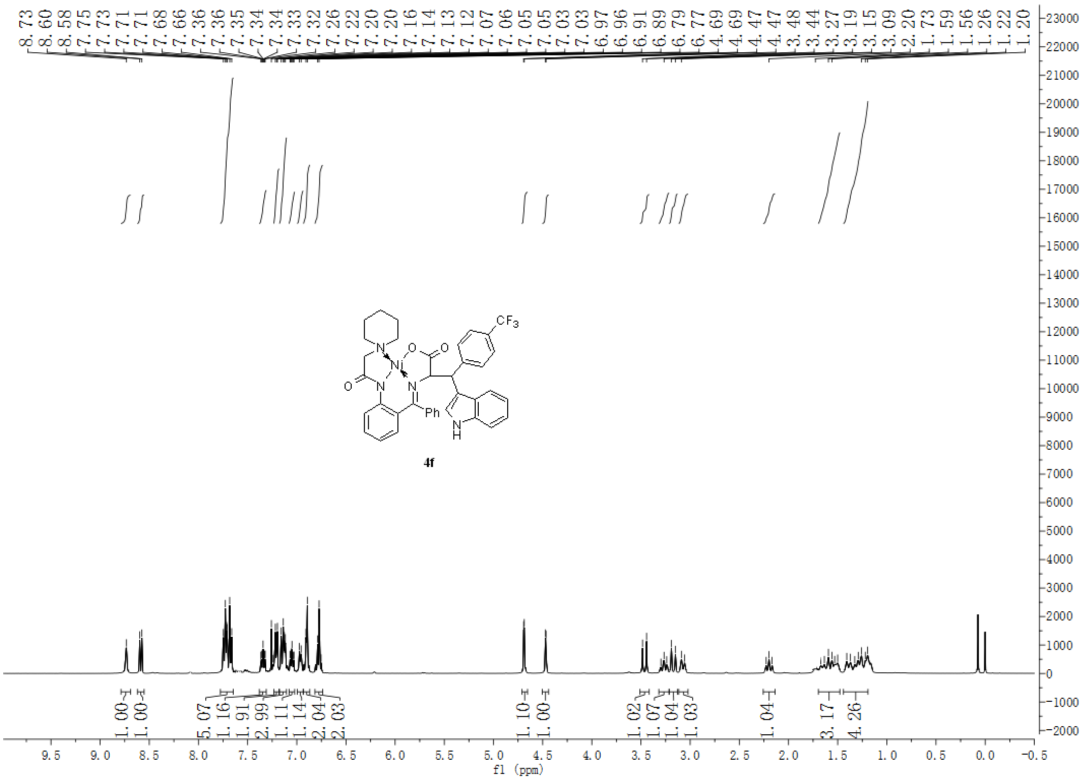

^{13}CNMR spectrum of compound **4f**

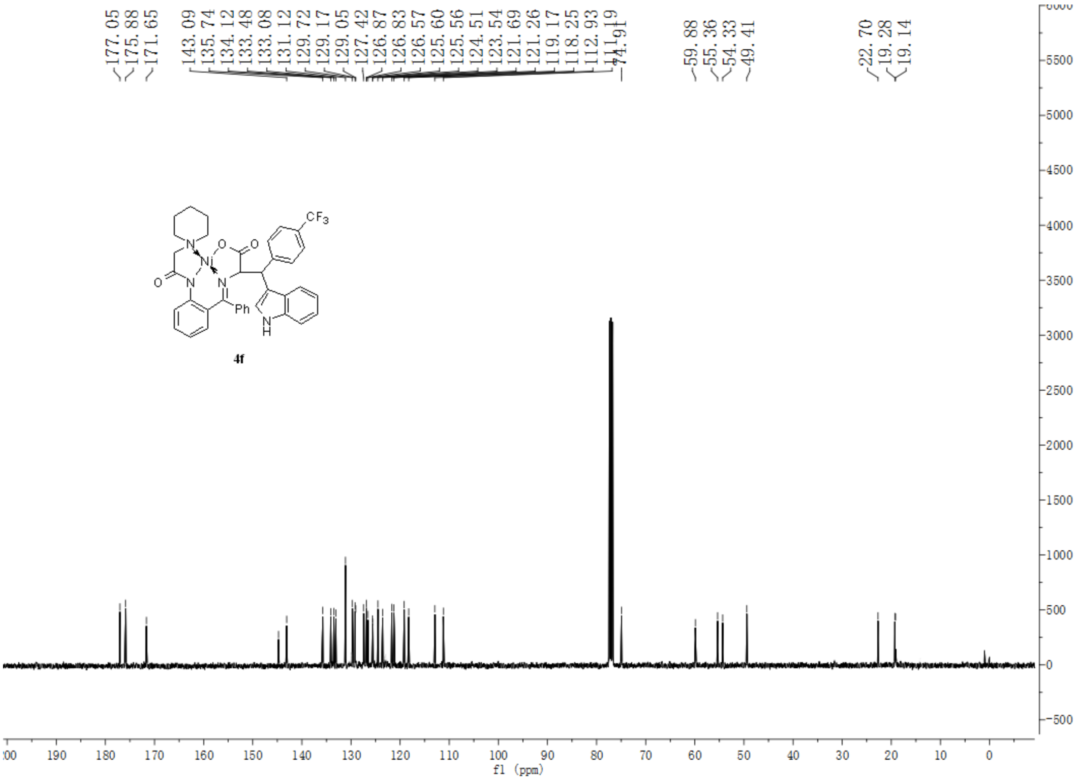

^{1}HNMR spectrum of compound **4g**

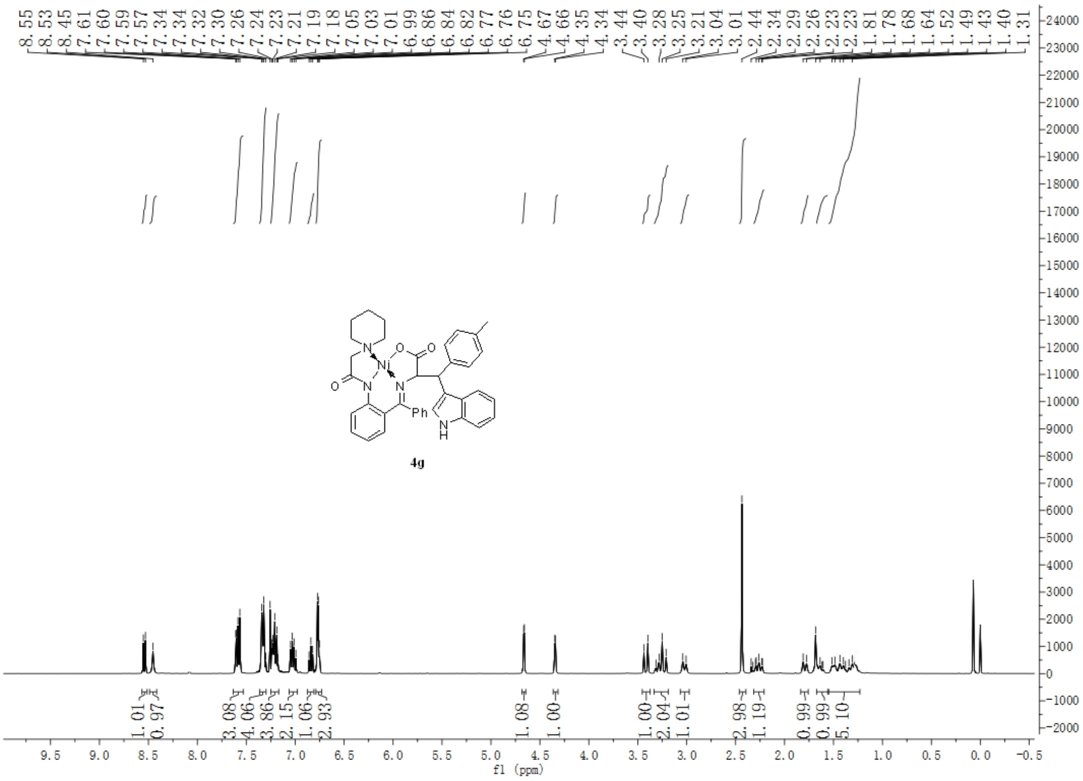

^{13}CNMR spectrum of compound **4g**

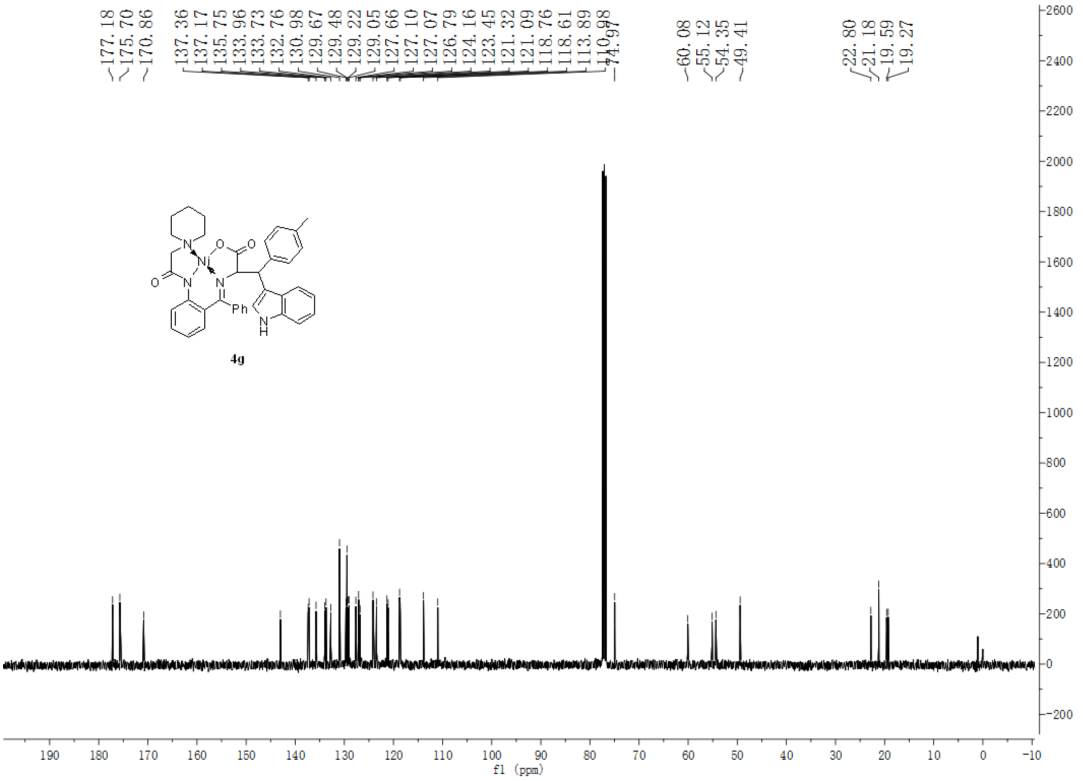

^{1}HNMR spectrum of compound **4h**

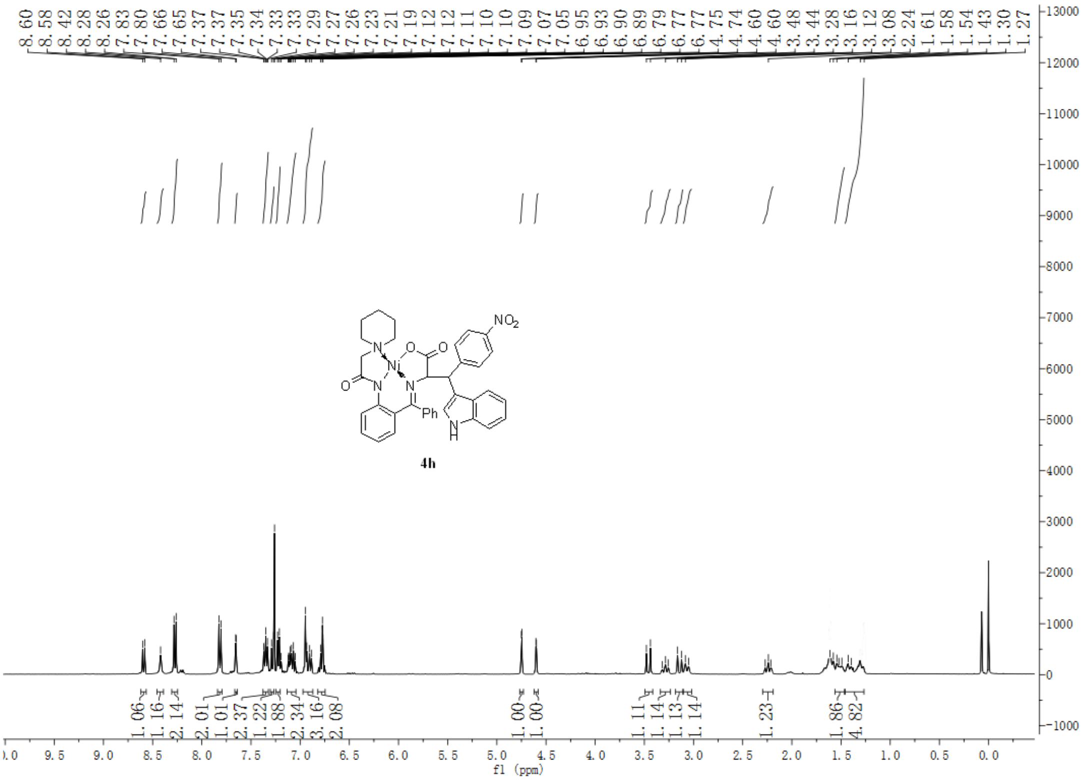

^{13}CNMR spectrum of compound **4h**

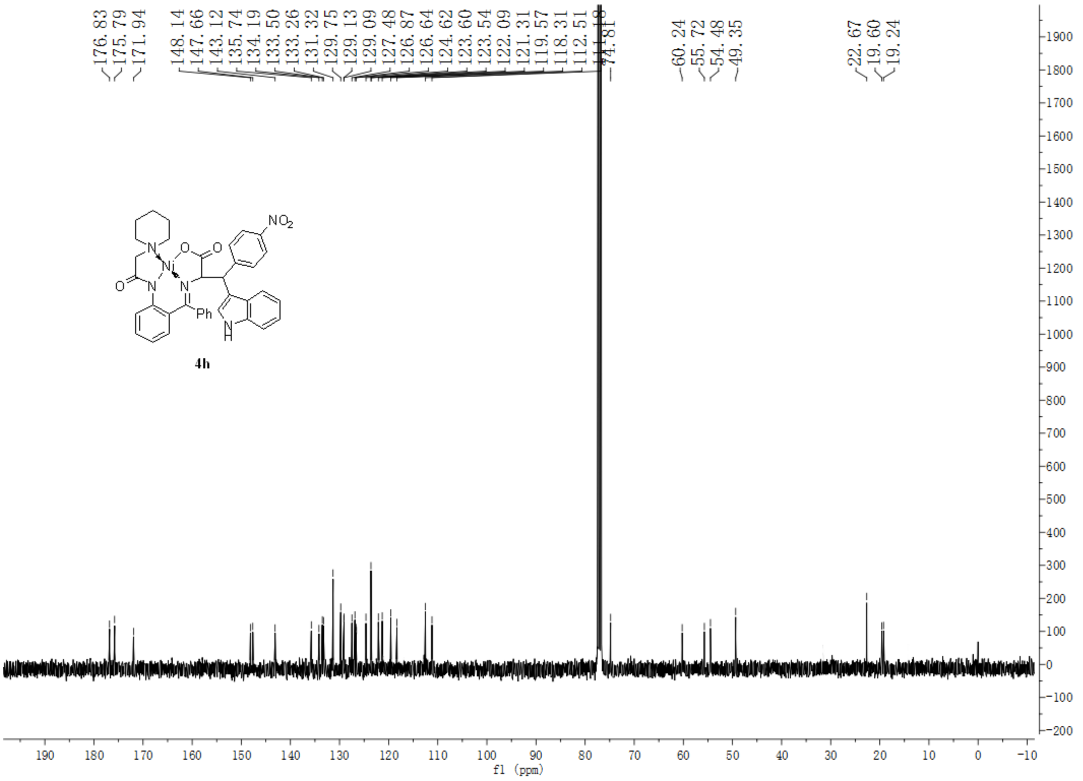

^{1}HNMR spectrum of compound **4i**

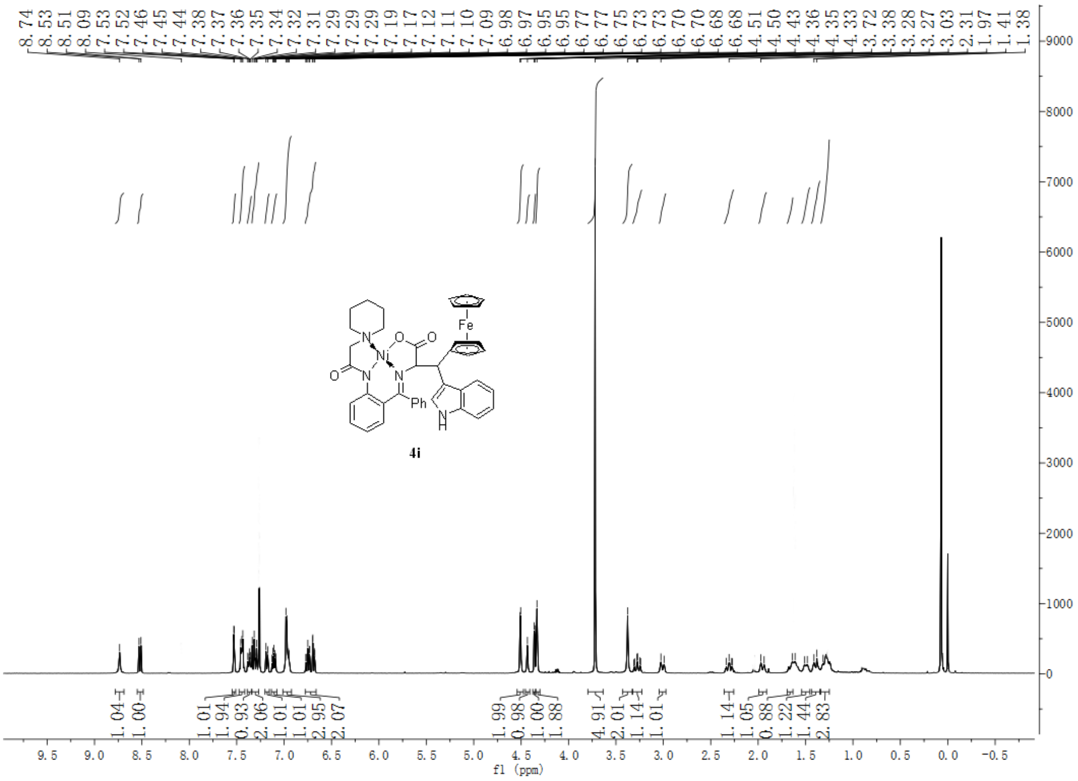

^{13}CNMR spectrum of compound **4i**

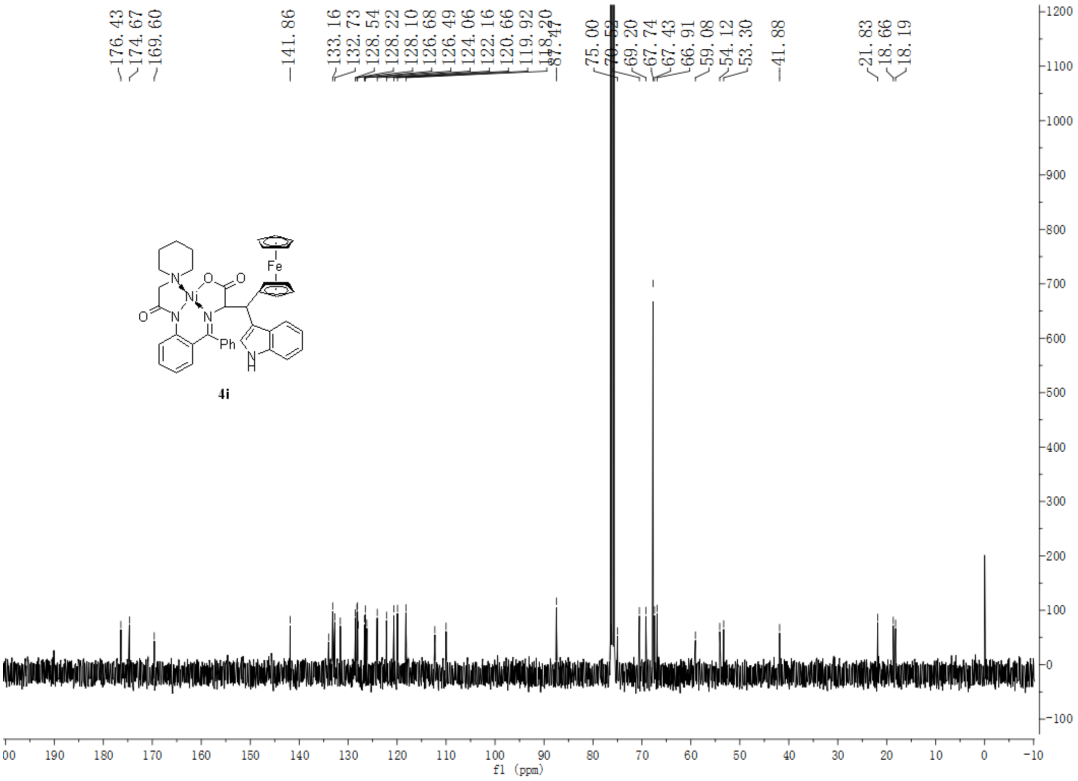

^{1}HNMR spectrum of compound **4j**

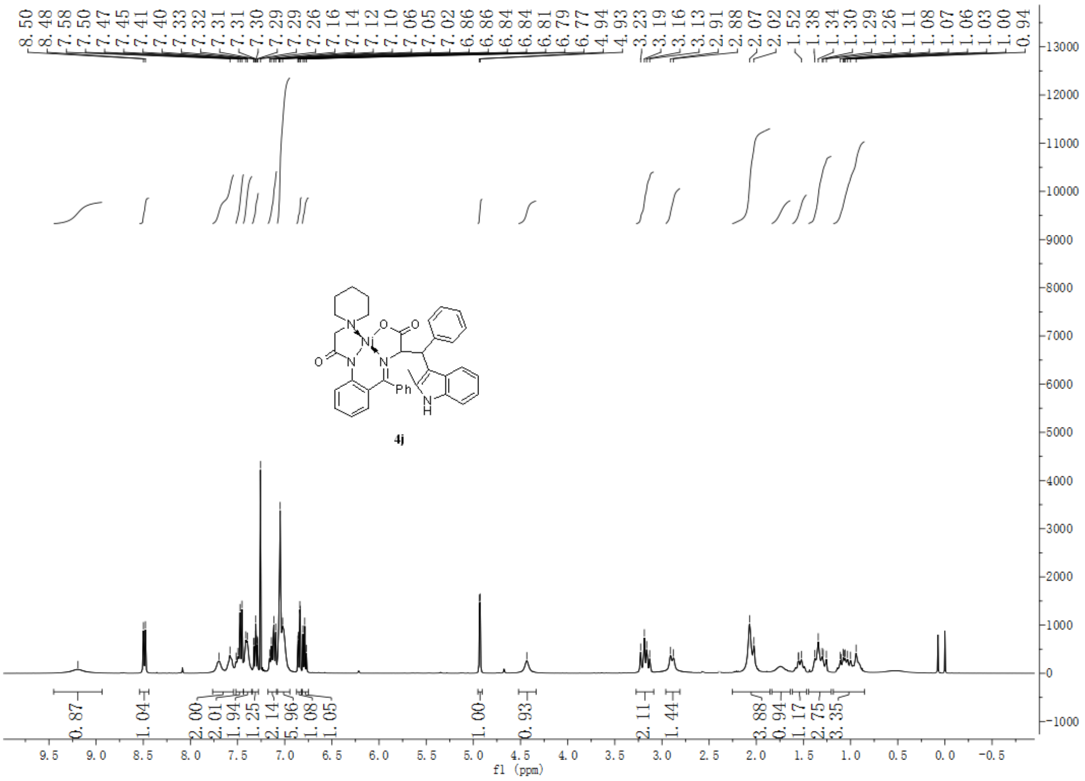

^{13}CNMR spectrum of compound **4j**

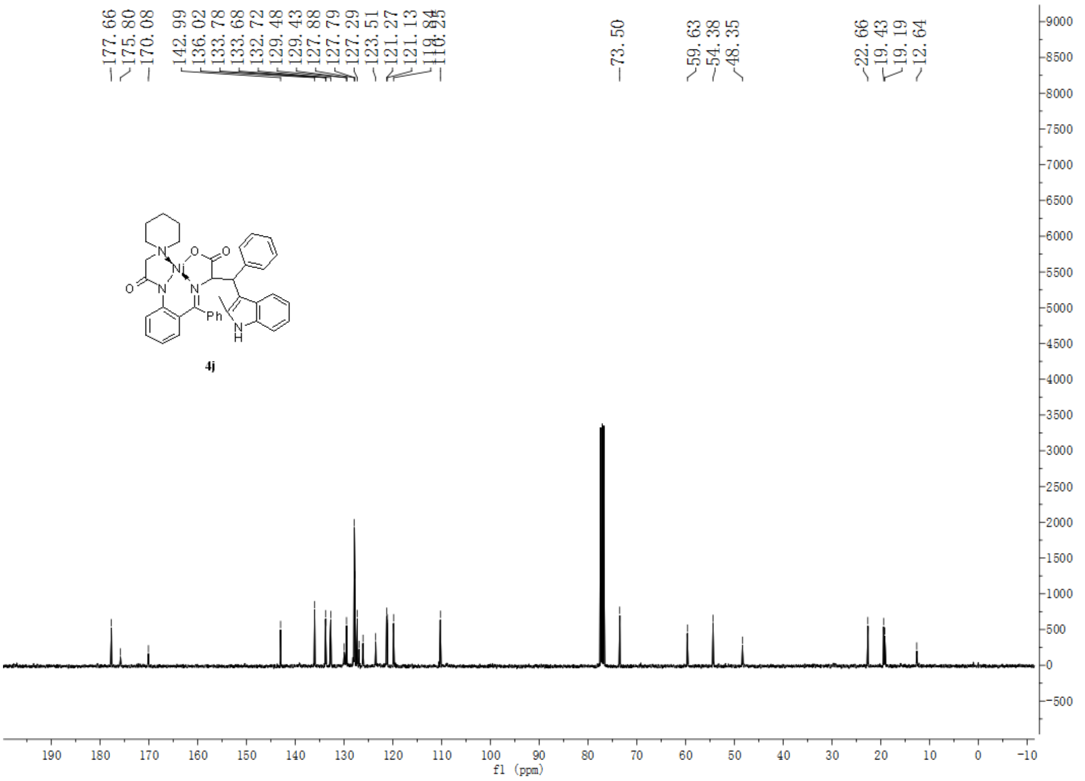

^{1}HNMR spectrum of compound **4k**

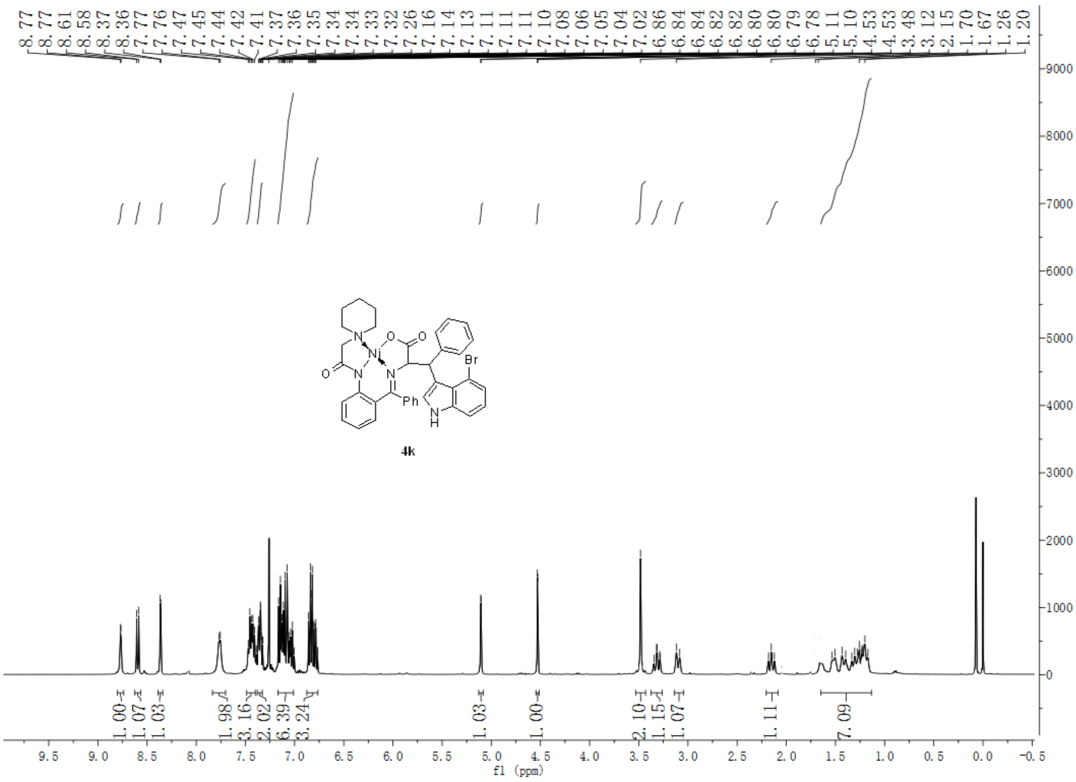

^{13}CNMR spectrum of compound **4k**

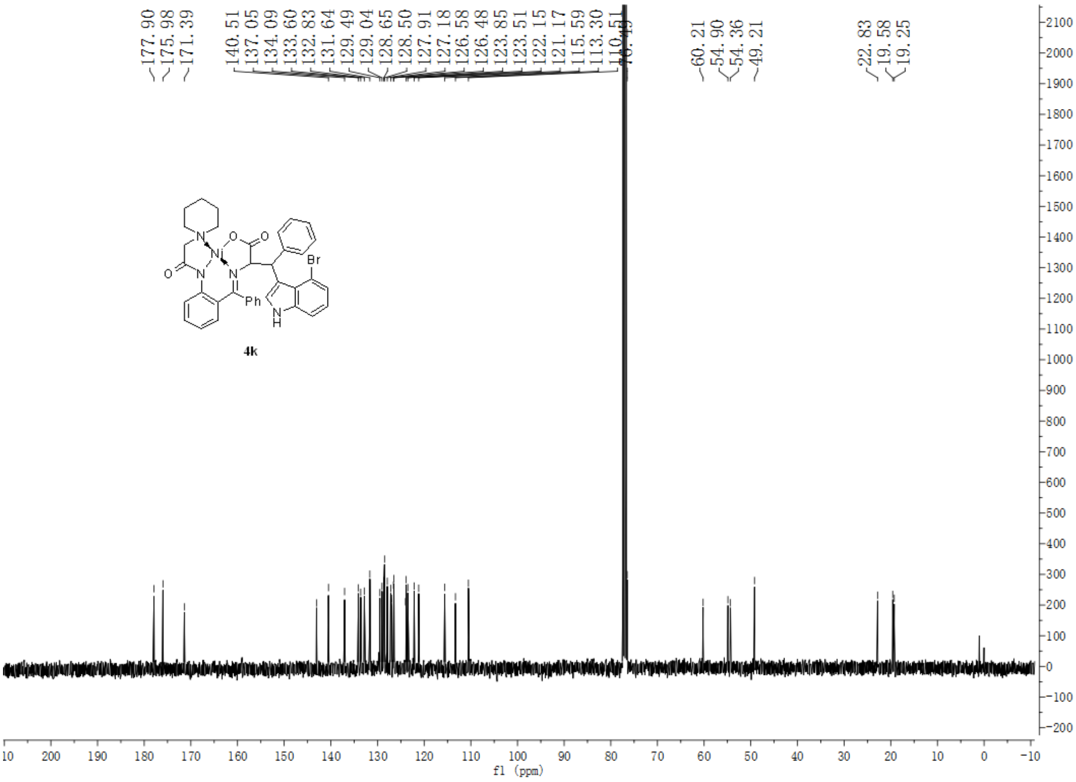

^{1}HNMR spectrum of compound **4l**

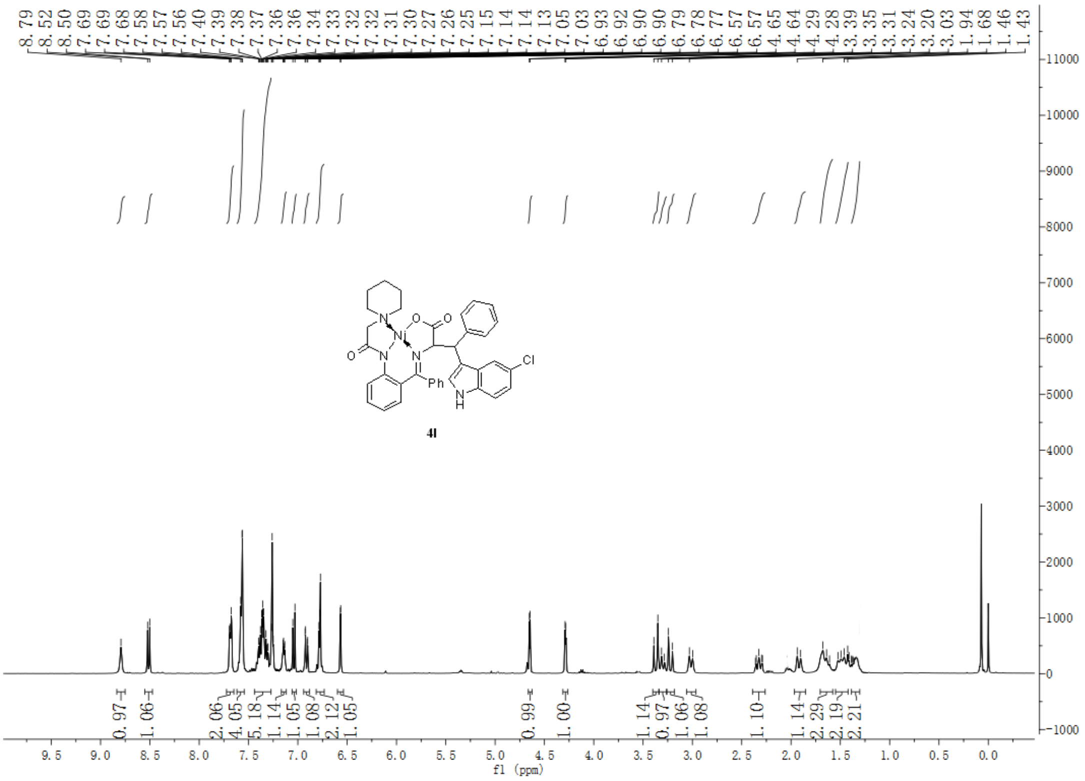

^{13}CNMR spectrum of compound **4l**

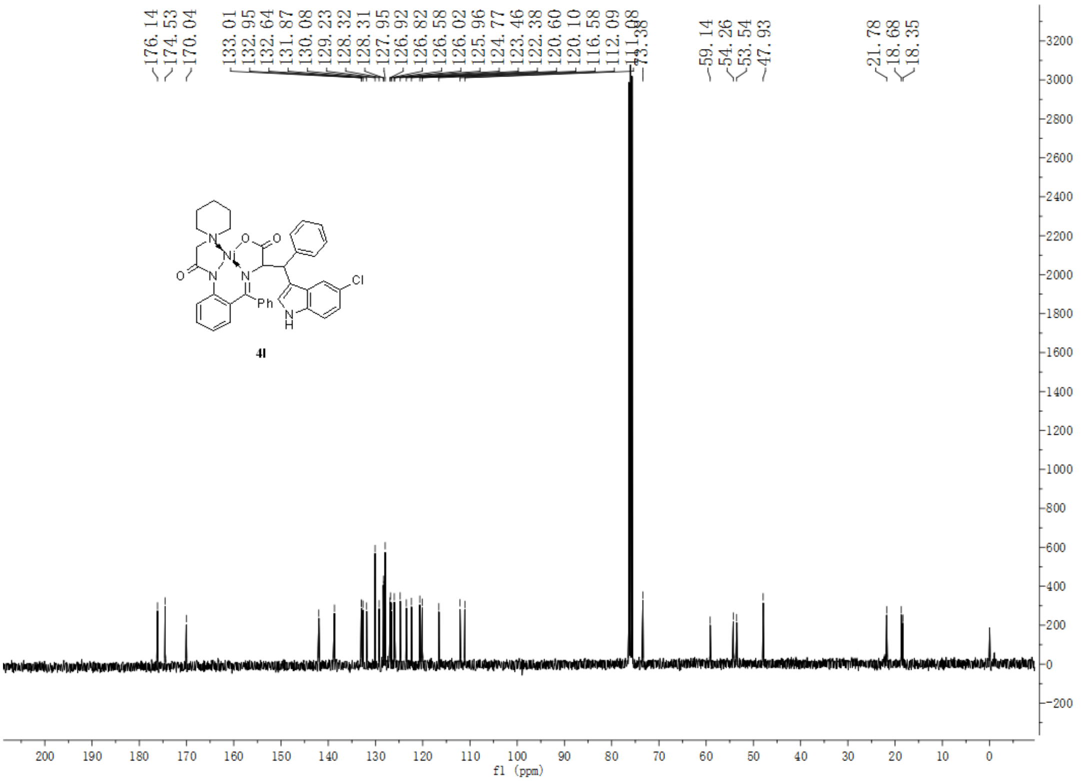

^{1}HNMR spectrum of compound **4m**

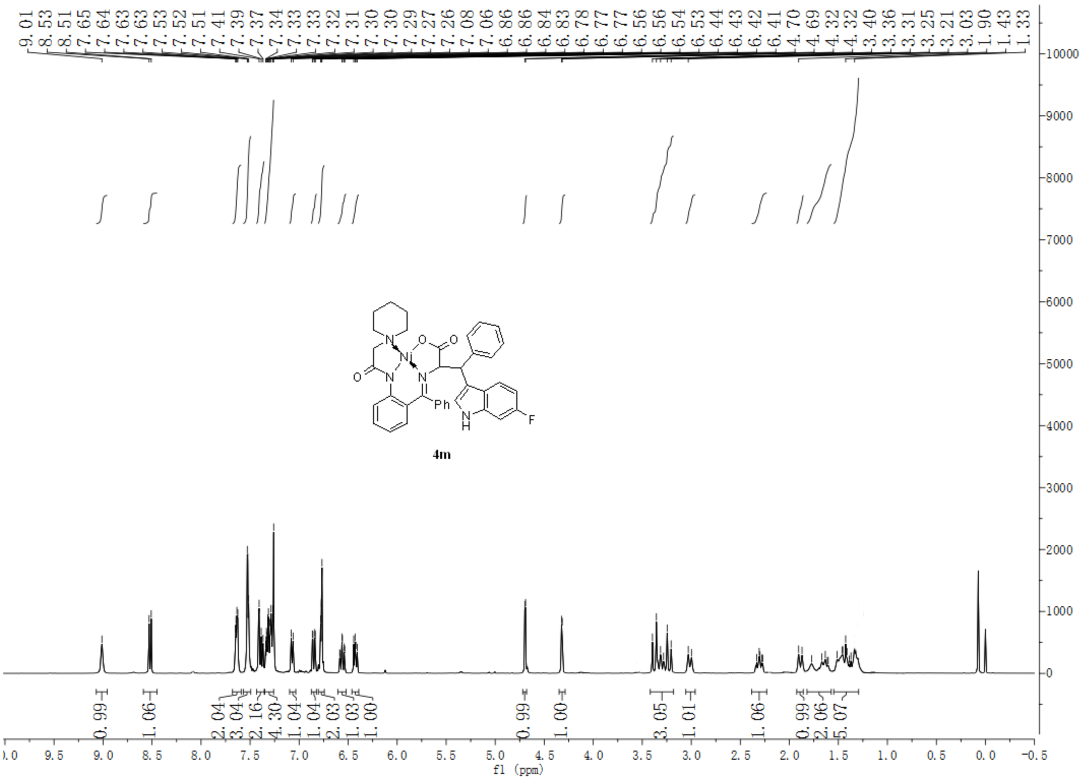

^{13}CNMR spectrum of compound **4m**

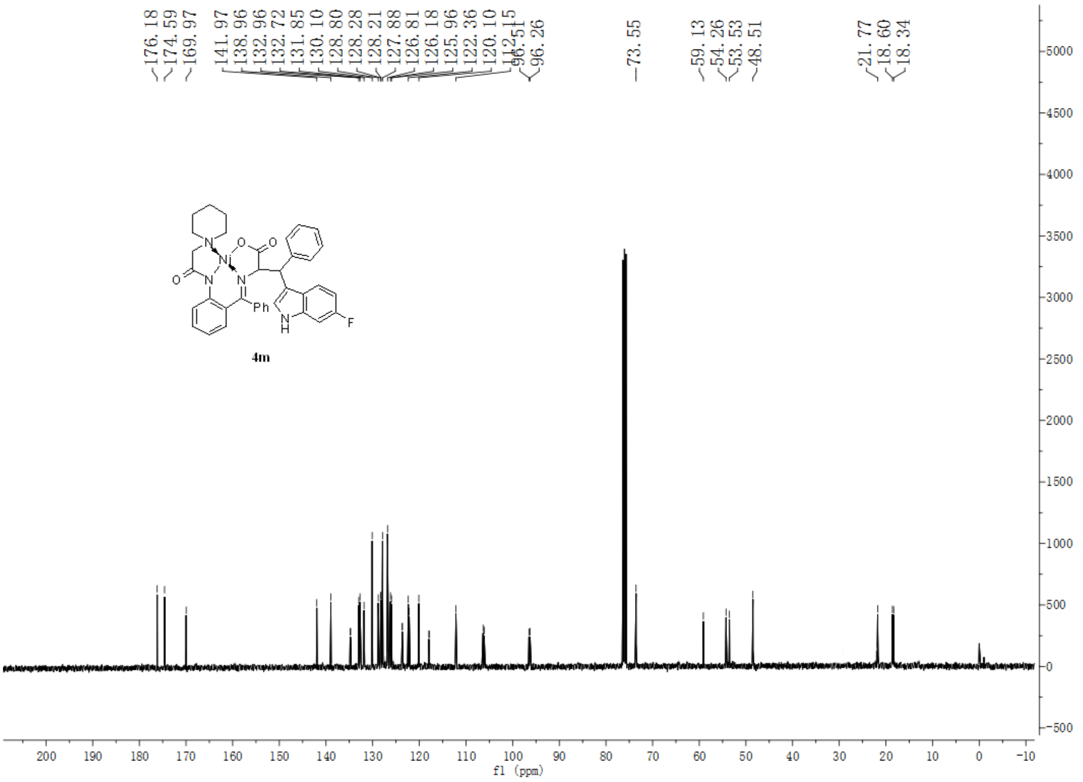

^{1}HNMR spectrum of compound **4n**

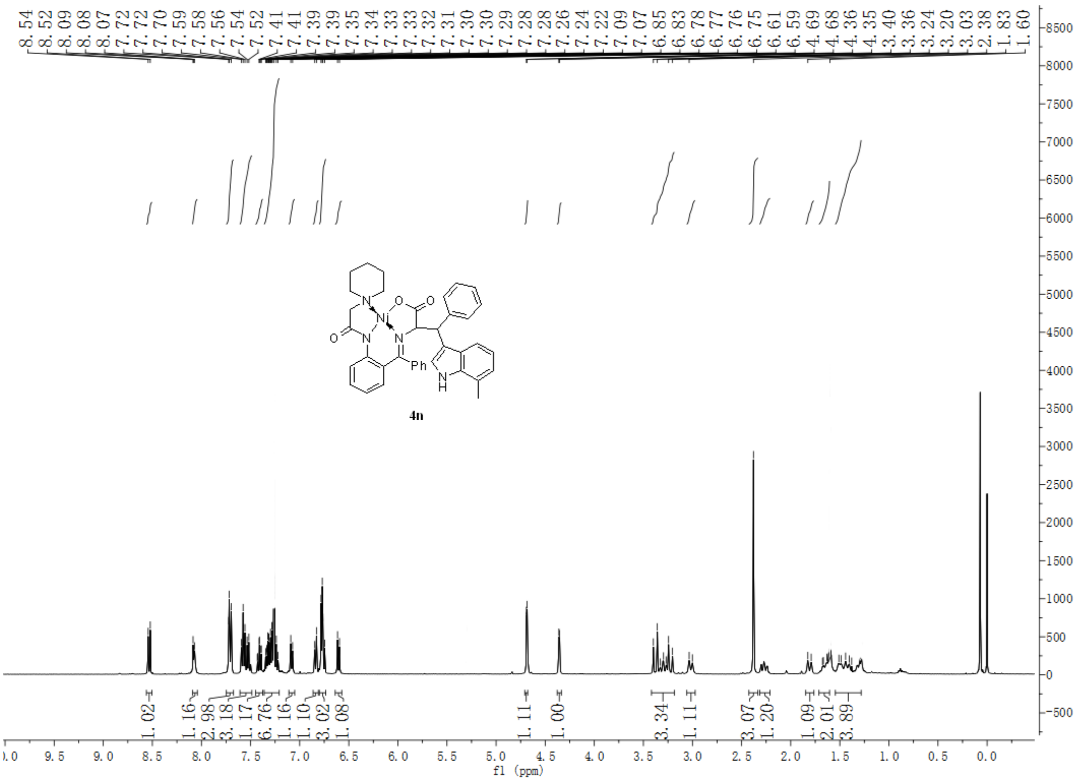

^{13}CNMR spectrum of compound **4n**

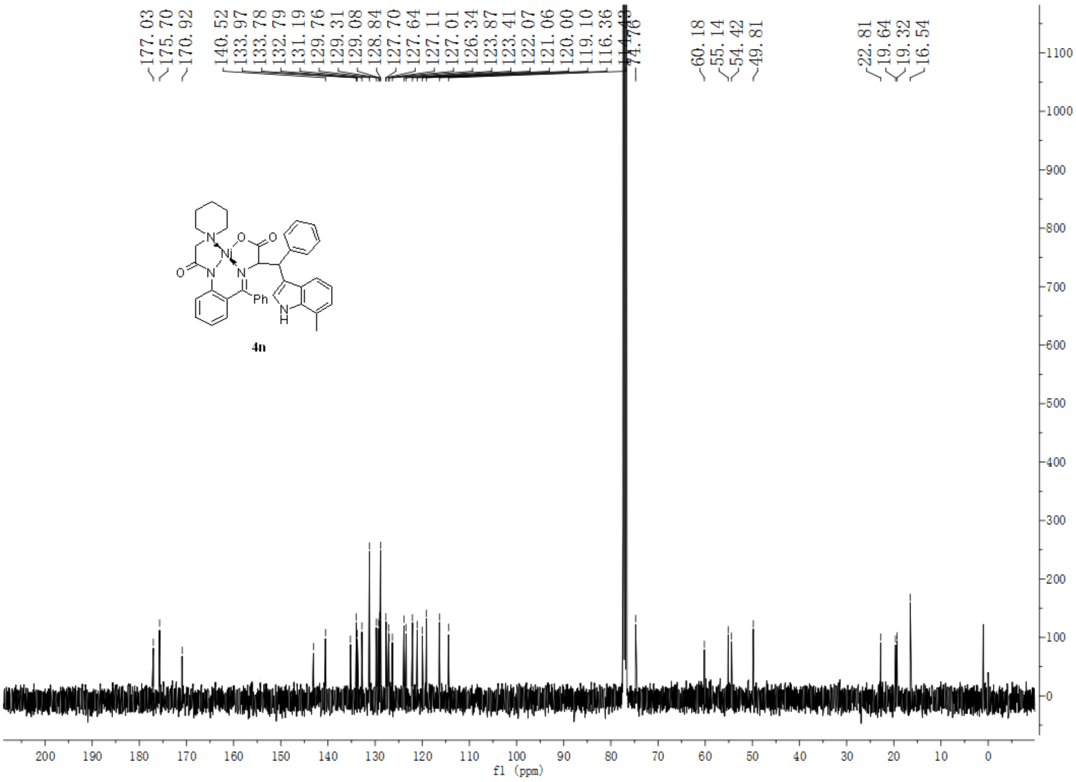

^{1}HNMR spectrum of compound **5a**

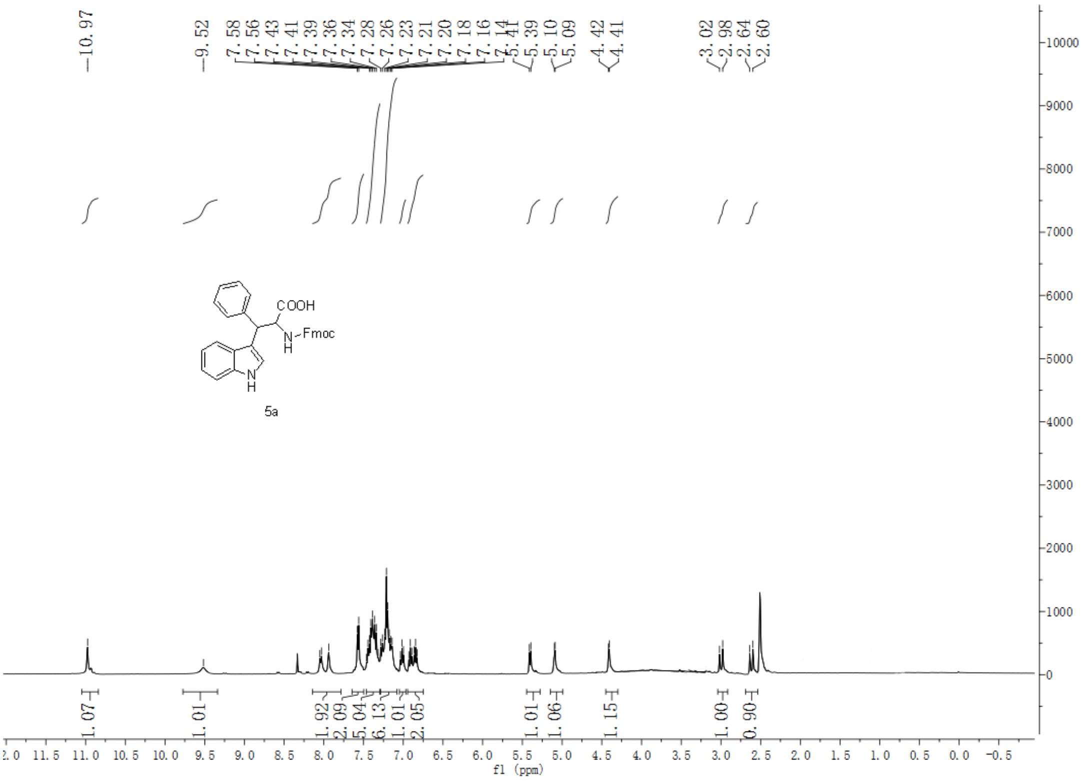

Supplement: Supplementary file 1 [file molecules-22-00695-s001.pdf]
